# Integrative taxonomy of *Metarhizium anisopliae* species complex, based on phylogenomics combined with morphometrics, metabolomics, and virulence data

**DOI:** 10.1186/s43008-024-00154-9

**Published:** 2024-09-11

**Authors:** Noppol Kobmoo, Suchada Mongkolsamrit, Artit Khonsanit, Marjorie Cedeño-Sanchez, Nuntanat Arnamnart, Wasana Noisripoom, Papichaya Kwantong, Chutima Sonthirod, Wirulda Pootakham, Alongkorn Amnuaykanjanasin, Esteban Charria-Girón, Marc Stadler, Janet Jennifer Luangsa-ard

**Affiliations:** 1 Integrative Crop Biotechnology and Management Research Group, Plant-Microbe Interaction Research Team, National Center for Genetic Engineering and Biotechnology (BIOTEC), National Science and Technology Development Agency (NSTDA), Pathum Thani, Thailand; 2 Department of Microbial Drugs, Helmholtz Centre for Infection Research GmbH (HZI), Inhoffenstraße 7, 6 38124 Braunschweig, Germany; 3 Institute of Microbiology, Technische Universität Braunschweig, Spielmannstraβe 7, Braunschweig, 38106 Germany; 4 Genomics Research Team, National Center for Genetic Engineering and Biotechnology (BIOTEC), National Omics Center, National Science and Technology Development Agency (NSTDA), Pathum Thani, Thailand; 5 Biorefinery and Bioproduct Technology Research Group, Biocontrol Technology Research Team, National Center for Genetic Engineering and Biotechnology (BIOTEC), National Science and Technology Development Agency (NSTDA), Pathum Thani, Thailand

**Keywords:** Integrative taxonomy, Population genomics, Virulence, Metabolomics, Morphology, Three new taxa

## Abstract

*Metarhizium anisopliae* (*Clavicipitaceae*, *Hypocreales*) is a globally distributed entomopathogenic fungus, which has been largely studied and used in agriculture for its potent entomopathogenicity. Since its taxonomic establishment as a member of *Metarhizium*, many closely related taxa have been described with highly similar morphology (cryptic species). A holotype specimen of *M. anisopliae* is not extant, and the ex-neotype strain (CBS 130.71) does not form a monophyletic clade with other strains, up to now, recognized as *M. anisopliae sensu stricto*. In this study, we have conducted an integrative taxonomic treatment of *M. anisopliae sensu lato* by including the ex-neotype strain of *M. anisopliae*, other unknown strains from our collections identified as *M. anisopliae s*. *lat*., as well as other known species that have been previously delimited as closely related but distinct to *M. anisopliae*. By including whole-genome sequencing, morphometric analysis, LC–MS based metabolomics, and virulence assays, we have demonstrated that *M. anisopliae s. str.* should also include *M. lepidiotae* (synonym), and that *M. anisopliae s. str.* differentiates from the other species of the complex by its metabolome and less severe entomopathogenicity. New taxa, namely *M. hybridum*, *M. neoanisopliae* and *M. parapingshaense* spp. nov., are proposed. The novel taxa proposed here have strong phylogenomics support, corroborated by fine-scale differences in the length/width of conidia/phialides, while the metabolomics and virulence data still largely overlap. We have also demonstrated via population genomics data the existence of local clonal lineages, particularly the one corresponding to the persistence of a biocontrol candidate strain that has been used in the field application for three years. This study showcases the utility of combining various data sources for accurate delimitation of species within an important group of fungal biocontrol agents against pest insects.

**Supplementary Information:**

The online version contains supplementary material available at 10.1186/s43008-024-00154-9.

## Introduction

*Metarhizium anisopliae* (*Clavicipitaceae*, *Hypocreales*) is a globally distributed species of entomopathogenic fungi widely used as a biocontrol agent against insect pests (Lubeck et al. 2008; Cao et al. 2016; Bamisile et al. 2018; Zhao 2023). This species was first discovered by Elias Metchnikoff (Metchnikoff 1879) as a pathogen of *Anisoplia austriaca* (*Coleoptera*) larvae in Ukraine (Zimmermann et al. 1995). Before the advent of molecular taxonomy, the nomenclature and classification of *M. anisopliae* had encountered numerous changes. The species was first described as *Entomophthora anisopliae *Metchnikoff (Metchnikoff 1879) before being transferred to *Metarhizium *based on molecular phylogenetics (Bischoff et al. 2009; Kepler et al. 2014). It was at times confused with and placed in *Penicillium* as *P. anisopliae* (Vuillemin 1904), and even with *Oospora* as *O. destructor* (Delacroix 1893; Aoki 1957; Kawakami 1960). Two major reasons for such confusion were that no original type material of Metchnikoff’s *M. anisopliae* was available, and molecular techniques were not used until the late twentieth century.

Veen (1968) typified *M. anisopliae *in a French-written thesis, and proposed a neotype culture (CBS 289.67), a proposal that was accepted by Tulloch (1976) in her revision of the genus. ARSEF 7487 was also derived from the original type strain (ex-neotype CBS 289.67), isolated from an Orthopteran insect from Eritrea (part of Ethiopia at that time), and deposited at the culture collection of the United States Department of Agriculture (USDA). This ex-neotype has been used as the reference strain in molecular taxonomic studies of *Metarhizium *during the twenty-first century (Driver et al. 2000; Bischoff et al. 2009; Kepler et al. 2014). It was thus not isolated from the same insect, nor the same locality as the original specimen of Metchnikoff. Mongkolsamrit et al. (2020) showed that the *M. anisopliae* strain CBS 130.71, isolated from the root of the cereal crop *Avena sativa* in Ukraine (the same locality as Metchnikoff’s original specimen) and thus designated as the new ex-neotype culture of *M. anisopliae*, is phylogenetically distant from ARSEF 7487 and closely related to *M. lepidiotae *(Bischoff et al. 2009). However, ARSEF 7487 still remains as *M. anisopliae*, which has left a situation where the ex-neotype culture of *M. anisopliae *(CBS 130.71) does not form a monophyletic clade with other specimens recognized as the same species, posing a contradiction to the phylogenetic species concept used for delimiting fungal species in the molecular era (Taylor et al. 2000).

Before the neotypification of *M. anisopliae *by Veen (1968), other *Metarhizium* species had been discovered, for example *M. album *(Petch 1931), *M. brunneum *(Petch 1935), and *M. flavoviride *(Gams and Rozsypal 1973), while Tulloch (1976) only accepted *M. anisopliae* and *M. flavoviride* as valid species. *Metarhizium guizhouense* and *M. pingshaense* were subsequently added to the genus in the late twentieth century (Guo et al. 1986). These species were shown to be closely related and many of them were occasionally considered to be only “varieties” within *M. anisopliae* or *M. flavoviride* (Driver et al. 2000). With molecular data, *M. anisopliae s. lat.* is considered to form a monophyletic clade, distinct from *M. flavoviride s. lat.* (*M. flavoviride* species complex) (Kepler et al. 2014; Mongkolsamrit et al. 2020). *Metarhizium anisopliae s. lat.* (*M. anisopliae* species complex) includes the PARB Clade (*M. anisopliae s. str.*, *M. brunneum*, *M. pingshaense*, and *M. robertsii*), the MGT Clade (*M. guizhouense* and *M. majus*), and also *M. lepidiotae*, *M. acridum* and *M. globosum* following Bischoff et al. (2009) and Kepler et al. (2014). Other recent species have been added to this complex mainly based on phylogenetic data, i.e. *M. kalasinense *(Luangsa-ard et al. 2017), *M. gryllidicola* and *M. phasmatodea *(Thanakitpipattana et al. 2020), *M. clavatum* and *M. sulphureum *(Mongkolsamrit et al. 2020). These recent studies have demonstrated the cryptic nature of *M. anisopliae s. lat.* where closely related species have very similar conidial shapes and sizes, whereas the classification of *Metarhizium *was mainly based on micro-morphological characters of asexual spores in the nineteenth and twentieth century (Sorokin 1883; Tulloch 1976). Despite the discovery of sexual morphs for some species (Sung et al. 2007; Mongkolsamrit et al. 2020), sexual characters (e.g. perithecial size, shape, and embedment; ascospore size and shape) have not been proven to be useful. Therefore, the identification of species within *M. anisopliae s. lat.* currently relies on molecular phylogenies.

The genus *Metarhizium *is also well-known for its capacity to infect a broad range of insects (St. Leger and Wang, 2020) and is believed to attack over 200 different host species (Veen 1968). It has been proposed that generalist species (PARB and MGT Clades) evolved from specialists as PARB and MGT Clades form a monophyletic clade branching from the most recent common ancestor shared with *M. acridum * which is specific to certain locusts and grasshoppers (Hu et al. 2014). The investigation on the variation of virulence and host specificity among intra-specific strains or strains from closely related species has been scarce. Previous studies focused on testing the virulence of a few strains, mainly from the PARB Clade known to infect a broad host range (Shapiro-Ilan 2009; Wang and Feng 2014; Lee 2019). The identification of *Metarhizium *isolates tested in these studies was not generally done in a taxonomically appropriate manner; either many strains were called “sp.” (Accoti et al. 2021) or putative “*M. anisopliae*” strains were identified as such based only on ITS data with broad morphological recognition (Dong et al. 2016). Therefore, the lack of an evolutionarily and taxonomically well-defined framework hinders a comprehensive understanding of the evolution of virulence as this trait cannot be directly linked to the most accurate and recent molecular phylogenies of this genus. Furthermore, the potential of virulence data to contribute to the taxonomy of *Metarhizium* has been overlooked. Evaluating virulence of various *Metarhizium* strains with a clear taxonomic classification will not only improve our comprehension of virulence, but also contribute to an integrative taxonomic elucidation based on virulence data.

It has been demonstrated that the virulence/pathogenicity of *Metarhizium *species is related to the production of secondary metabolites (Rohlfs and Churchill 2011). Particularly, various destruxins produced by *Metarhizium *were shown to have insecticidal effects (Pal et al. 2007; Donzelli et al. 2012). Previous chemotaxonomic work has shown more quantitative difference in the production of secondary metabolites between distantly related species than closely related fungal species (Kobmoo et al. 2021; Wongkanoun et al. 2023), in line with the idea of cryptic species being recognizable only by nucleotide data. Recently, Barelli et al. (2022) have shown that the production pattern of destruxins is different between four *Metarhizium* species, three of which are from *M. anisopliae s. lat.* (i.e. *M. acridum*, *M. brunneum,* and *M. robertsii*) and one from *M. flavoviride*. However, there are more species to explore (Mongkolsamrit et al. 2020) and the lack of comprehensive metabolomics data prevents us from testing the hypothesis whether the production pattern of destruxins can be explained by evolutionary history.

Fungal cryptic species are increasingly being discovered thanks to whole-genome sequencing (WGS) data (Passer et al. 2019; Maharachchikumbura et al. 2021; Kobmoo et al. 2021). Combined with other sources of data such as morphological, metabolomics, and ecological data, WGS enables the elucidation of species status among closely related taxa (Seehausen et al. 2014), an approach called “integrative” or “holistic taxonomy” (Grube et al. 2017; Boluda et al. 2019; Stengel 2022). Abundant genomic resources available today for *Metarhizium *(Pattemore et al. 2014; Prasad et al. 2015; Wang et al. 2016) have been exploited principally for understanding the molecular basis to virulence and pathogenicity (Hu et al. 2014), but less to apprehend natural genetic variations (but see Rizal et al. 2024). Not only has WGS contributed to recent taxonomic discoveries, but this technique can also be used for tracking fungal strains of interest in field applications (Mei et al. 2020; Peng et al. 2021).

In this study, we have used WGS to identify and validate new species status for unknown strains from our old collections along with recent collections from an agricultural area where a candidate strain of *Metarhizium *(BCC 4849; Wasuwan et al. 2022) was released. The main objective of this study is to resolve species status within *M. anisopliae* species complex. We examined (1) genomic diversity, (2) phenotypic variation on conidia and phialides features, (3) variation in secondary metabolite production, and (4) virulence towards an insect species (*Spodoptera exigua*, *Lepidoptera*), for species within *M. anisopliae s. lat*. By including Tulloch’s ex-neotype *M. anisopliae* strain (ARSEF 7487) and the recent ex-neotype culture of *M. anisopliae s. str. *proposed by Mongkolsamrit et al. (2020) (CBS 130.71), the various sources of data have been combined under an integrative taxonomic framework to propose three new species, namely *M. neoanisopliae*, *M. hybridum*, *M. parapingshaense*, and to synonymize *M. lepidiotae* with *M. anisopliae s. str*. *Metarhizium neoanisopliae* BCC 4849 is shown here to persist in the agricultural area where it was applied to control insect pests.

## Materials and methods

### Sample collection

Unidentified strains of *Metarhizium* spp. were ordered from the BIOTEC Culture Collection (BCC) (Table 1). These had been collected during numerous excursions around Thailand by the research team at BIOTEC. They were selected for this study based on our in-house taxonomic identification (ITS barcoding) which showed their affiliation to *M. anisopliae s. lat*. Most were isolated from mycosed insects following Mongkolsamrit et al. (2020); asexual spores (conidia) from the fresh specimens were harvested with a flame-sterilized needle and streaked on a potato dextrose agar (PDA) plate (PDA: freshly diced potato 200 g/L, dextrose 20 g/L, agar 15 g/L), incubated at room temperature for 24 h, then checked for contamination. Uncontaminated germinating spores were transferred to another PDA plate for incubation at 25 °C to get pure cultures before being stored at -80 °C in liquid nitrogen at the BCC. The specimens were dried and deposited at the BIOTEC Bangkok Herbarium (BBH). A few strains were recently isolated by the BIOTEC Biocontrol Technology Research Team from the soil of a fruit orchard (Table 1), where the strain BCC 4849 was used as a biocontrol agent. The soil-borne isolates were obtained using a streak plate method with the following protocol: Soil samples each weighing approx. 500 g were collected from a depth of 10–15 cm using a trowel, removing litter. The samples were then placed in plastic bags and transported to the laboratory. Fungal isolation was carried out within two days of collection using a dilution plating technique and cetyltrimethyl ammonium bromide (CTAB) and oatmeal agar (OA) as a basal medium following the procedure described in Abdullah et al. (2015).

**Table 1.** List of *Metarhizium* spp. included in this study (^T^ = ex-type strain, ^M^ = included in the morphometric analysis, ^C^ = included in the metabolomics analysis, ^V^ = included in the virulence assays). Culture collection and fungarium codes: *BBH* BIOTEC Bangkok Herbarium, *BCC* BIOTEC Culture Collection, *CBS* Fungal and yeast collection of WI-KNAW (CBS-KNAW) Culture Collection, *ARSEF* U.S. Department of Agriculture (USDA) Culture Collection

**Sample code**	**Genus**	**Species**	**Location **	**host**	**Culture Collection**	**Herbarium**	**Isolation** **Date**
CBS 130.71^T,M,C,V^	*Metarhizium*	*anisopliae*	Ukraine	*Avena sativa* (Plant)	CBS 130.71 = ATCC 22269	CBS H-14432	–
ARSEF 7488^M,C^	*Metarhizium*	*anisopliae* (=*lepidiotae*)	Australia	*Coleoptera*	ARSEF 7488	–	–
ARSEF 7412^M,C,V^	*Metarhizium*	*anisopliae* (=*lepidiotae*)	Australia	*Coleoptera*	ARSEF 7412	–	–
ARSEF 2080^M,C,V^	*Metarhizium*	*neoanisopliae* sp. nov.	Indonesia	*Nilaparvata lugens* (*Hemiptera, Delphacidae*)	ARSEF 2080	–	
PGP239.1 (SM2416)	*Metarhizium*	*neoanisopliae* sp. nov.	Thailand	Soil	BCC 96583	–	16-Nov-21
ARSEF 7487^T,M,C,V^	*Metarhizium*	*neoanisopliae* sp. nov.	Ethiopia	*Orthoptera*	ARSEF 7487 = CBS 289.67 = IMI 168777	CBS H-7330	–
ARSEF 7450^M,C^	*Metarhizium*	*neoanisopliae* sp. nov.	Australia	*Coleoptera*	ARSEF 7450	–	–
BCC4849^M,C,V^	*Metarhizium*	*neoanisopliae* sp. nov.	Thailand	Treehole Materials	BCC 4849	–	28-Sep-98
CP1-S24.1 (SM2387)	*Metarhizium*	*neoanisopliae* sp. nov.	Thailand	Soil	BCC 96565	–	16-Nov-21
CP2-S100 (SM2402)^V^	*Metarhizium*	*neoanisopliae* sp. nov.	Thailand	Soil	BCC 96580	–	16-Nov-21
CP2-S77.1 (SM2398)	*Metarhizium*	*neoanisopliae* sp. nov.	Thailand	Soil	BCC 96576	–	16-Nov-21
CP2-S99.1 (SM2400)^M^	*Metarhizium*	*neoanisopliae* sp. nov.	Thailand	Soil	BCC 96578	–	16-Nov-21
CP1-S38.1 (SM2393)	*Metarhizium*	*neoanisopliae* sp. nov.	Thailand	Soil	BCC 96571	–	16-Nov-21
PGP247.1 (SM2414)^M,C,V^	*Metarhizium*	*neoanisopliae* sp. nov.	Thailand	Soil	BCC 96581	–	16-Nov-21
ARSEF 549^M,C,V^	*Metarhizium*	*hybridum* sp. nov.	Brazil	-	ARSEF 549	BBH 50656	–
ARSEF 3210^M,C,V^	*Metarhizium*	*pingshaense*	India	*Coleoptera*	ARSEF 3210	–	––
CBS 257.90^T,C,M^	*Metarhizium*	*pingshaense*	China	*Coleoptera*	CBS 257.90	–	
CP1-S36.1 (SM2391)	*Metarhizium*	*pingshaense*	Thailand	Soil	BCC 96569	–	16-Nov-21
CP2-S66 (SM2397)^M,C^	*Metarhizium*	*pingshaense*	Thailand	Soil	BCC 96575	–	16-Nov-21
CP2-S10 (SM2395)	*Metarhizium*	*pingshaense*	Thailand	Soil	BCC 96573	–	16-Nov-21
MY12337^M,V^	*Metarhizium*	*pingshaense*	Thailand	*Dermaptera*	BCC 93000	–	24-Jun-20
ARSEF 7929^C,V^	*Metarhizium*	*pingshaense*	Australia	*Isoptera*	ARSEF 7929	–	–
ARSEF 4342^M,C,V^	*Metarhizium*	*parapingshaense* sp. nov.	Solomon Islands	*Coleoptera*	ARSEF 4342	–	–
PGP252.1 (SM2415)^M,V^	*Metarhizium*	*parapingshaense* sp. nov.	Thailand	Soil	BCC 96582	–	16-Nov-21
MY5150^M,C^	*Metarhizium*	*parapingshaense* sp. nov.	Thailand	Insect (*Diptera*)	BCC 37941	BBH 26560	16-Aug-09
ARSEF 4739^C^	*Metarhizium*	*robertsii*	Australia	Soil	ARSEF 4739	–	29-Feb-88
ARSEF 727^C^	*Metarhizium*	*robertsii*	Brazil	*Orthoptera*	ARSEF 727	–	–
ARSEF 8820^C^	*Metarhizium*	*robertsii*	USA	*Coleoptera: Curculionidae*	ARSEF 8820	–	–
ARSEF 2107^T,C^	*Metarhizium*	*brunneum*	USA	*Coleoptera*	ARSEF 2107	–	–
MY11578^T,C^	*Metarhizium*	*gryllidicola*	Thailand	adult crickets	BCC 82988	BBH 44436	1-Nov-16
MY7483	*Metarhizium*	*gryllidicola*	Thailand	*Orthoptera*	BCC 53857	BBH 32733	11-Jul-12
NHJ11527^C^	*Metarhizium*	*gryllidicola*	Thailand	*Orthoptera*	BCC 12817	BBH 8383	18-Aug-01
MY3226	*Metarhizium*	*gryllidicola*	Thailand	adult of crickets	BCC 30917	BBH 23876	18-Jun-08
MY5073	*Metarhizium*	*gryllidicola*	Thailand	adult of crickets	BCC 37915	BBH 26529	14-Aug-09
MY1341^C^	*Metarhizium*	*gryllidicola*	Thailand	adult of crickets	BCC 22353	BBH 18647	5-Jul-06
MY5085^C^	*Metarhizium*	*gryllidicola*	Thailand	adult of crickets	BCC 37918	BBH 26533	15-Aug-09
MY11637^T,C^	*Metarhizium*	*clavatum*	Thailand	*Oxynopterus* sp. (*Coleoptera*),	BCC 84543	BBH 43330	30-May-17
MY11677^C^	*Metarhizium*	*clavatum*	Thailand	*Oxynopterus *sp. (*Coleoptera*)	BCC 84558	BBH 42806	28-Jun-17
MY0008^C^	*Metarhizium*	*clavatum*	Thailand	Insect (*Dictyoptera*)	BCC 16474	BBH 10006	13-Jul-2004
NHJ10822^C^	*Metarhizium*	*phasmatodea*	Thailand	*Coleoptera* larva	BCC 2841	–	–
MY6900^T,C^	*Metarhizium*	*phasmatodea*	Thailand	*Orthoptera*: *Phasmatodea*	BCC 49272	BBH 32532	16-Aug-11
MY7343^C^	*Metarhizium*	*kalasinense*	Thailand	*Coleoptera* larva	BCC 53581	BBH 34584	15-Jun-12
MY7440^C^	*Metarhizium*	*kalasinense*	Thailand	*Coleoptera* larva	BCC 53629	BBH 32209	26-Jun-12
ARSEF 6238^C^	*Metarhizium*	*guizhouense*	China	*Lepidoptera*	ARSEF 6238	–	–
CBS258.90^C^	*Metarhizium*	*guizhouense*	China	*Lepidoptera*	CBS 258.90	–	–
NHJ11819^C^	*Metarhizium*	*sulphureum*	Thailand	*Lepidoptera* larva	BCC 12791	–	–
NHJ14124^C^	*Metarhizium*	*sulphureum*	Thailand	–	BCC 18130	–	–
MY5321	*Metarhizium*	*sulphureum*	Thailand	*Lepidoptera* larva	BCC 39045	BBH 27261	13-Sep-09
MY4581^T,C^	*Metarhizium*	*sulphureum*	Thailand	*Lepidoptera* larva	BCC 36592	BBH 29463	21-May-09
MY4549	*Metarhizium*	*sulphureum*	Thailand	*Lepidoptera* larva	BCC 36575	BBH 27054	19-May-09
MY6023	*Metarhizium*	*sulphureum*	Thailand	*Lepidoptera* larva	BCC 42068	BBH 28572	1-Jun-10
MY4504	*Metarhizium*	*sulphureum*	Thailand	*Lepidoptera* larva	BCC 36547	BBH 26174	12-May-09
MY4561	*Metarhizium*	*sulphureum*	Thailand	*Lepidoptera* larva	BCC 36585	BBH 26213	20-May-09
MY4542^C^	*Metarhizium*	*sulphureum*	Thailand	*Lepidoptera* larva	BCC 36568	BBH 26200	19-May-09
MY4543	*Metarhizium*	*sulphureum*	Thailand	*Lepidoptera* larva	BCC 36569	BBH 26201	19-May-09
MY4547	*Metarhizium*	*sulphureum*	Thailand	*Lepidoptera* larva	BCC 36573	BBH 26205	19-May-09
MY4552	*Metarhizium*	*sulphureum*	Thailand	*Lepidoptera* larva	BCC 36578	BBH 26209	19-May-09
MY4545^C^	*Metarhizium*	*sulphureum*	Thailand	*Lepidoptera* larva	BCC 36571	BBH 26203	19-May-09
MY4548^C^	*Metarhizium*	*sulphureum*	Thailand	*Lepidoptera* larva	BCC 36574	BBH 26206	19-May-09
MY4376	*Metarhizium*	*sulphureum*	Thailand	*Lepidoptera* larva	BCC 36280	BBH 26103	24-Apr-09
MY4541	*Metarhizium*	*sulphureum*	Thailand	*Lepidoptera* larva	BCC 36567	BBH 26199	19-May-09
MY4546^C^	*Metarhizium*	*sulphureum*	Thailand	*Lepidoptera* larva	BCC 36572	BBH 26204	19-May-09
ARSEF 1914^T,C^	*Metarhizium*	*majus*	Philippines	*Coleoptera*	ARSEF 1914	–	–
ARSEF 1015^C^	*Metarhizium*	*majus*	Japan	*Lepidoptera*	ARSEF 1015	–	–
ARSEF 2133^T,C^	*Metarhizium*	*flavoviride*	Czech Republic	*Coleoptera*	ARSEF 2133	–	–
CBS 700.74^C^	*Metarhizium*	*flavoviride*	USA	–	CBS 700.74	–	–
ARSEF 4124^T,C^	*Metarhizium*	*frigidum*	Australia	*Coleoptera*	ARSEF 4124	–	–
ARSEF 324	*Metarhizium*	*acridum*	Australia	*Orthoptera*	ARSEF 324	–	–
ARSEF 7486^T,C^	*Metarhizium*	*acridum*	Niger	*Orthoptera*	ARSEF 7486	–	–
ARSEF 2596^T,C^	*Metarhizium*	*globosum*	India	*Lepidoptera*	ARSEF 2596	–	–

Other strains, shown in previous studies (Kepler et al. 2014; Luangsa-ard et al. 2017; Mongkolsamrit et al. 2020; Thanakitpipattana et al. 2020) to represent different species within *M. anisopliae s. lat.* (i.e. *M. acridum*, *M. brunneum*, *M. clavatum*, *M. globosum*, *M. guizhouense*, *M. kalasinense*, *M. phasmatodea*, *M. pingshaense*, *M. robertsii*, *M. sulphureum*, and *Metarhizium* sp.) were ordered from the CBS-KNAW and USDA collections. These included the ex-neotype culture of *M. anisopliae s. str. *(CBS 130.71) designated by Mongkolsamrit et al. (2020), and the ex-neotype culture of *M. anisopliae *(ARSEF 7487) sensu Tulloch (1976) and re-affirmed by Kepler et al. (2014).

### DNA extraction and whole-genome sequencing

The cultures were grown on PDA plates. A 1 × 1 cm^2^ of agar per strain was cut to be put on another plate for incubation at 25 °C for one to two weeks. DNA extraction was done using the CTAB-based procedure described in Kobmoo et al. (2021). The genomic DNA was checked for purity and quantity with a Nanodrop spectrophotometer (Thermo Fisher) and 1% agar gel electrophoresis. For WGS, approximately 500 ng of genomic DNA was used for library construction following the MGIEasy FS DNA Library Prep Kit (MGI Tech, Shenzhen, China). Paired-end sequencing (150 bp) was performed using the MGISEQ-2000RS Sequencing Flow Cell V3.0 on the DNASEQ-400 according to the manufacturer’s protocol.

### Population genomics and phylogenomics analyses

MegaBOLT version 1.5.6.11 (MGI) was used to extract the sample barcode and to control the quality of the raw read data of each sample. The controlled reads were mapped on the reference genome of *M. anisopliae *JEF-290 (Lee 2019), using bwa v0.7.17-r1188 (Li and Durbin, 2009) with default settings. The single nucleotide polymorphisms (SNPs) were identified by GATK v4.1.4.1 with HaplotypeCaller function (McKenna et al. 2010). The SNPs were filtered to possess the following features: 1,000 < all-sample depth < 10,000, mapping quality > 40, quality normalized by depth > 20, Fisher strand bias < 10 and strand odd ratio < 3. The individual genotypes with depth < 10 and genotyping quality < 20 were marked as missing data. The filtering was based on the a posteriori distribution of the features. Only the SNPs with no missing data and minor allele frequency (MAF) > 0.05 were retained, resulting in a total of 98,085 SNPs.

An initial snapshot of the diversity of *Metarhizium *spp. included in this study was obtained by an F84 distance-based neighbor-joining tree, inferred directly from the SNPs data. The population structure was inferred with a Bayesian clustering analysis implemented via the software FastStructure (Raj et al. 2014). Genotypes were then classified into multi-locus genotypes (MLG) to represent clonal groups. This classification was done using the R package *poppr *(Kamvar et al. 2014) following the method of Grünwald and Hoheisel (2006). To construct a phylogenomic tree, we first extracted gene sequences with nucleotides altered by SNPs using *FastaAlternateReferenceMaker *from the GATK toolbox (McKenna et al. 2010). This extraction was based on the gene model associated to the reference genome of *M. anisopliae *JEF-290 (Lee 2019). Positions with indels were marked as missing data. The identity of the single-copy genes was determined via the web server OrthoVenn2 (Xu et al. 2019). Only single-copy genes with a minimum length of 500 bp and a ratio of SNPs/nucleotide greater than 0.005 were retained. Such criteria ensured finding at least two SNPs for each gene alignment to guarantee a minimum level of phylogenetic signal, resulting in 237 genes. The gene sequences from different samples were aligned using MAFFT (Katoh and Standley 2013). Subsequently, gene-wise alignments were concatenated, resulting in a final alignment of 451,019 bp. The best maximum likelihood (ML)-based phylogenomic tree was constructed using IQ-TREE (Nguyen et al. 2015). Branch supports were determined with 1,000 replicates of ultrafast bootstrap (Hoang et al. 2018). The best sequence evolution model (TVM + I + G4) was inferred using ModelTest-NG (Darriba et al. 2020). The final tree was rooted with the *M. flavoviride* complex (*M. flavoviride* and *M. frigidum*). Additionally, a coalescent-based species tree was also estimated using ASTRAL-III (Zhang et al. 2018). This was done to consider potential conflicts between gene trees, which could introduce a systemic bias in the concatenated gene trees (Liu et al. 2015).

### Morphological characterization

The macro-morphological characters and relevant data of the fungi, including the host, were examined under a dissecting microscope (Olympus SZ61). For micro-morphological characterization, phialides and conidia were mounted on a microscope slide with a drop of lactophenol cotton blue solution and measured using a compound microscope (Olympus CX31). The length and the width of conidia and phialides from pure cultures were measured for representative strains from the novel taxa (Table 1). For *M. pingshaense* and *M. parapingshaense*, conidia were also collected from dried specimens and measured for length and width. These data were subjected to an analysis of variance (ANOVA) (Kaufmann & Schering 2014) testing for the difference between species with the strains included as a random factor. Thirty phialides and conidia per strain were measured. The fungal cultures were grown on PDA and incubated at 25 °C under a light/dark cycle (L:D = 14:10). The cultures were observed for comparison of essential morphological characters, including the shapes and sizes of conidia, phialides, colony growth, and coloration.

For the descriptions, the color of specimens and cultures incubated on PDA was codified following the Royal Horticultural Society’s Colour Chart (Royal 2015).

### Metabolomics

Strains (Table 1) were grown in 200 mL of yeast malt extract broth (YM broth; malt extract 10 g/L, yeast extract 4 g/L, D‐glucose 4 g/L, pH 6.3 before autoclaving) at 23 °C under shaking condition (140 rpm). Each culture was inoculated with 5 pieces of mycelia (7 mm diam) from a fully grown YM agar plate. Due to the growth rate differences among strains, the prolongation of each culture after glucose depletion was set to be half of the time required for that particular strain to reach glucose depletion. This method is justified as exhaustion of the first limiting nutrient influences the growth curve and should be considered for experimental standardization (Vrabl 2019). The glucose content during the culturing was estimated using urine glucose test strips (DIRUI®, Jilin, China).

The extraction of secondary metabolites was performed according to Phainuphong et al. (2017). The mycelia were separated from the culture broth via vacuum filtration and the mycelial yield was determined. The culture broth was extracted twice with equal amount of ethyl acetate (v/v). The resulting organic phase was filtered through anhydrous sodium sulphate and evaporated on a rotary evaporator to dryness. The mycelia were soaked in acetone and extracted twice under sonication (30 min). The solvent was evaporated to yield an aqueous phase that was extracted twice with ethyl acetate, in a similar manner to the culture broth. The samples (Table 1) were processed in two batches separated by a year due to experimental limitations.

Each sample was analyzed using the instrumental settings and conditions reported previously (Charria-Girón et al. 2023). Raw data were pre-processed with MetaboScape 2022 (Bruker Daltonics, Bremen, Germany) in the retention time range of 0.5–25 min, and the obtained features were dereplicated based on their accurate molecular weight and MS/MS spectra against the compounds reported from *Metarhizium *spp. in the Natural Product Atlas (NP Atlas) database (Van Santen et al. 2019). For this purpose, MetaboScape performed automatic in silico MS/MS matching based on the InChI-encoded structures using the MetFrag algorithm in the absence of MS/MS reference data (Ruttkies et al. 2016).

The metabolomics analysis was conducted using the data of peak area. As the extracts were prepared in two batches, to remove any systematic bias due to separate batch processes, any feature absent in either batch was discarded and the raw data from the respective batch were normalized within the batch before merging the whole data. A principal component analysis (PCA) was conducted to detect any pattern corresponding to divergence between taxa. The analysis was conducted separately between the data from BE and CE. The data of annotated compounds were extracted to construct a heatmap based on Manhattan distance using the gplots package v.3.0.1 (Warnes et al. 2016) in R (R Core Team 2020).

### Virulence assays

The virulence assays were conducted on beet armyworms (*Spodoptera exigua*, *Lepidoptera*). To assess interspecific variation in virulence, representative strains from species within PARB and *M. anisopliae s. str.* (Table 1) were subcultured on PDA at 25 °C for varying periods (1 week–1 month) until sporulation. These strains were selected as they represent the proposed novel species (*M. hybridum*, *M. neoanisopliae,* and *M. parapingshaense*) along with closely related known species (*M. anisopliae s. str.*, *M. pingshaense,* and *M. robertsii*). For the assays on beet armyworms, spores from each strain were harvested into 1 ml of sterilized water, counted, and the concentration adjusted to 10^8^ spores/ml. Afterwards, 3 μl of the spore suspension was injected into each beet armyworm. Thirty beet armyworms, divided into three replicates of ten individuals, were injected with the spore suspension. The mortality of the insects was monitored daily for one week. Two types of mortality data were considered: (1) unconditional mortality, i.e. any insect found dead was considered as non-survival, and (2) mortality with mycelia, i.e. only insects found dead and covered with fungal mycelia were considered as non-survival. This distinction aimed to disentangle different fungal fitness components as the fungi would be considered as having successfully reproduced only if they managed to develop through the insects’ body to produce spores for further generations. The mortality rate was calculated for each replicate as the number of dead insects with or without covering fungal mycelia divided by the total number of insects per replicate (10).

To analyze the mortality rate data, a linear model was fitted using generalized least squares, i.e. gls command from the package nlme in R (R Core Team 2020), accounting for the correlation between the observations within replicates nested within strains. The species and days of observations were considered as interacting fixed effects and were tested using ANOVA.

## Results

### Population structure and clonal diversity

**Fig. 1:**
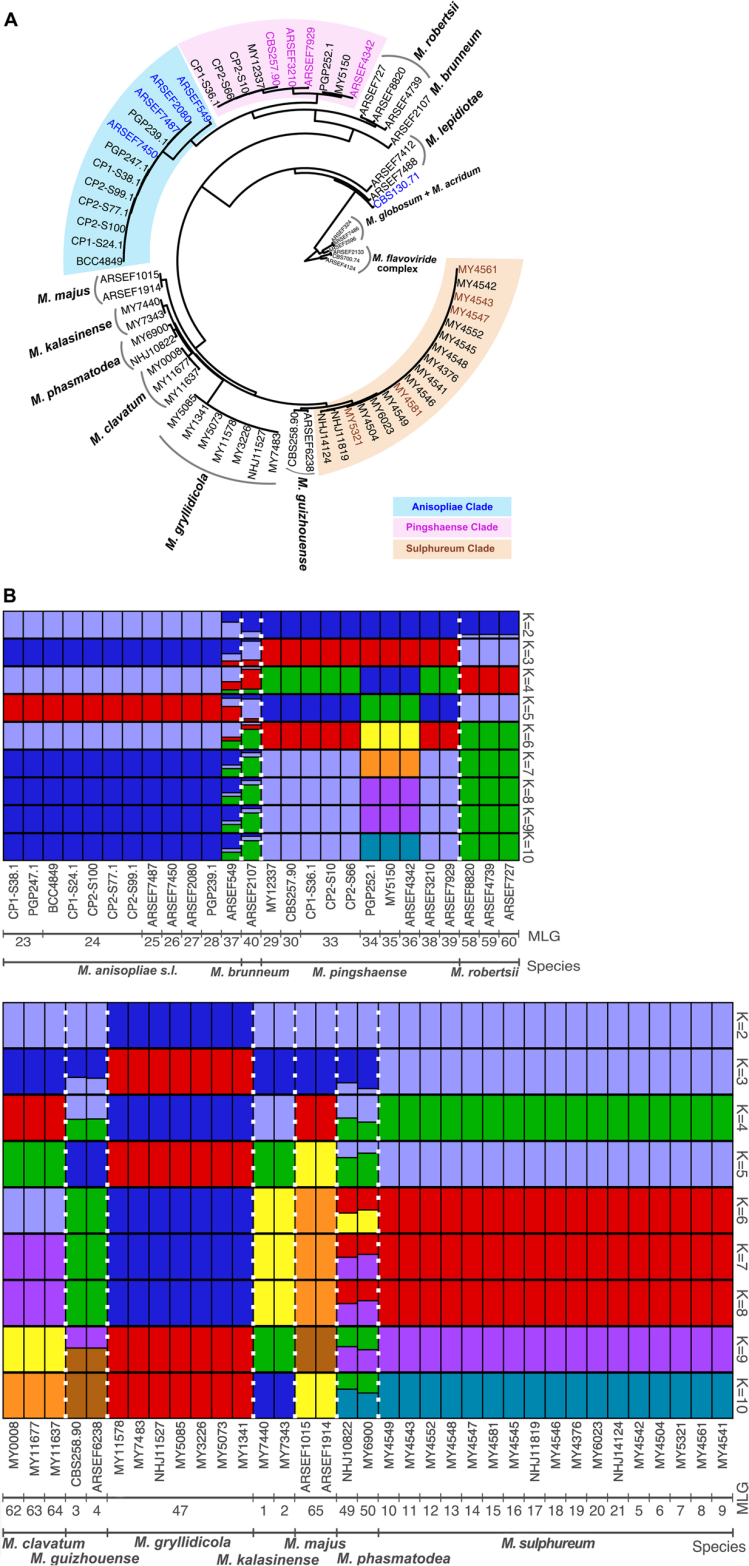
Results of population genomics analyses based on 98,085 SNPs. **A** F84 distance-based neighbor-joining tree showing the identification of unknown strains among three species complexes; colored labels represent strains previously recognized as respective species, *M. anisopliae* (blue), *M. pingshaense* (pink) and *M. sulphureum* (brown). **B** Bayesian clustering analysis focusing on the PARB group (upper panel), and MGT group (lower panel); *MLG *Multi-Locus Genotype (clonal group)

The F84 distance-based neighbor-joining tree constructed from 98,085 SNPs revealed that the unidentified strains clustered with three *Metarhizium* species, *M. anisopliae*, *M. pingshaense,* and *M. sulphureum* (Fig. 1A). The *M. anisopliae* clade (blue clade, Fig. 1A) comprises known strains of what had been previously identified as *M. anisopliae s. lat.* (ARSEF 549) and *M. anisopliae s. str.* (ARSEF 2080, ARSEF 7487, ARSEF 7450) in the catalog of ARS Collection of Entomopathogenic Fungal Cultures (ARSEF). Surprisingly, the ex-neotype strain of *M. anisopliae s. str.* (CBS 130.71) did not cluster with this clade but branched as a basal taxon close to *M. lepidiotae*. Other unknown strains from our collections clustered either with known strains of *M. pingshaense* (pink clade, Fig. 1A), or *M. sulphureum* (brown clade, Fig. 1A). Other strains of known species each formed a monophyletic clade, corresponding to their distinctive taxonomic status.

**Fig. 2:**
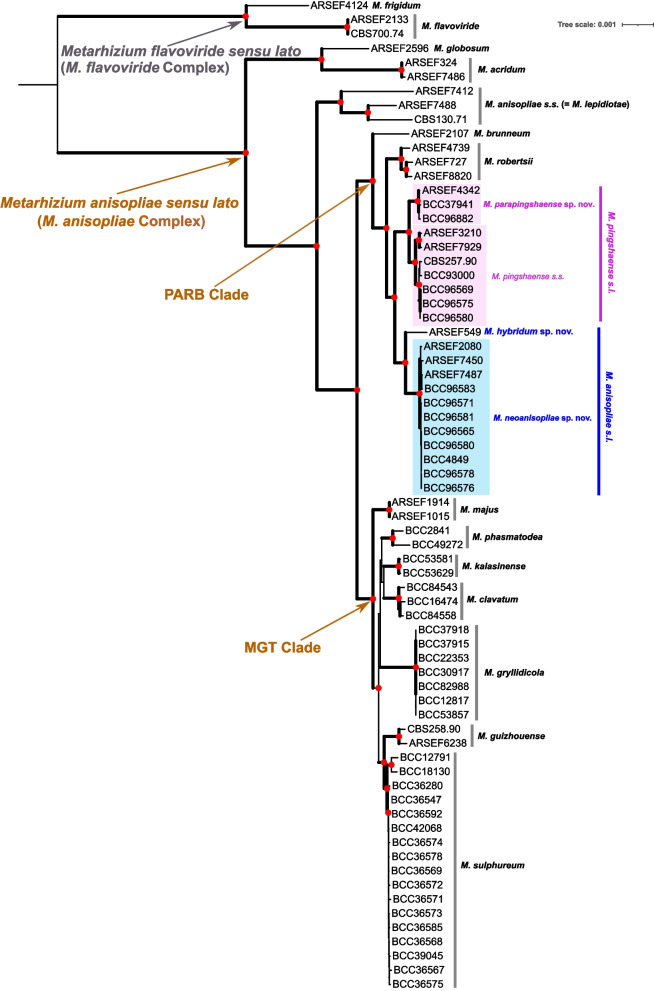
A ML-based phylogenomic tree from a concatenated matrix of 237 genes harnessed across the genome. Thick branches are supported by 100% bootstrapped, the red dots represent the nodes with 100% posterior probability following a coalescent-based species tree (Additional File 2: Figure S1)

We further conducted Bayesian clustering analyses focusing on the PARB group (*M. anisopliae* clade, *M. pingshaense* clade, *M. robertsii*, and *M. brunneum*: Fig. 1B, upper panel) and MGT group (*M. sulphureum* clade, *M. guizhouense*, *M. gryllidicola*, *M. clavatum*, *M. phasmatodea*, *M. kalasinense,* and *M. majus*: Fig. 1B, lower panel). The analyses confirmed the genetic differentiation between different species but revealed also strains/species which might have originated from hybridization; ARSEF 549, clearly showed a genetic mixed ancestry, probably between *M. anisopliae s. lat.* (= *M. neoanisopliae*) and *M. robertsii* or *M. brunneum*; ARSEF 2107, the type strain of *M. brunneum*, with the dominant genetic origin from *M. robertsii. Metarhizium phasmatodea* within the MGT group also exhibited a signal of mixed ancestry, probably between *M. sulphureum* and *M. kalasinense*/*M. clavatum*. There are two distinct genetic groups within the *M. pingshaense* clade (Fig. 2B, upper panel).

The 70 strains included in this study could be classified into 55 multi-locus genotypes (MLG), i.e. clonal groups (Additional File 1: Table S1). Besides ARSEF 549 with its mixed genetic ancestry, thus constituting on its own a clonal group (MLG 37), the *M. anisopliae *clade comprised six MLG (23–28). *Metarhizium pingshaense *clade is composed of eight MLG (29, 30, 33–36, 38, 39). *Metarhizium gryllidicola* and *M. majus* were respectively composed of a unique clonal lineage (MLG 47 for *M. gryllidicola* and 65 for *M. majus*) while all the remaining species were composed of multiple MLG, each represented by a single strain. Overall, this showed that some species were probably genetically more diverse than others. It is to be noted that the biopesticide strain BCC 4849 is part of MLG 24, represented also by four other strains (SM2387 = BCC96565, SM2402 = BCC96580, SM2398 = BCC96576, SM2400 = BCC96578) isolated from the soil of the same agricultural field.

### Phylogenomics species tree

**Fig. 3:**
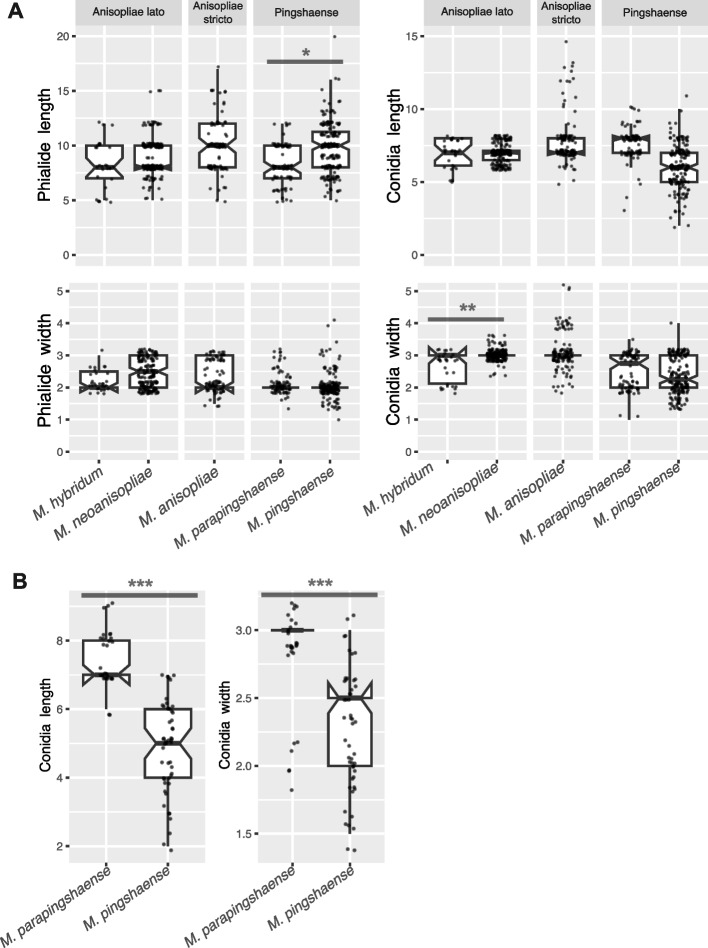
Notched boxplots representing the distribution of the width and the length (μm) of phialides and conidia between the novel taxa and closely related species from **(A)** cultures, and **(B)** stromatal samples. Statistical significance (one-factor ANOVA): * = *p* < 0.05, *** = *p* < 0.001

We constructed an ML-based phylogenomic tree from a matrix of 237 genes, partitioned to accommodate the best-fitted model for each gene (Fig. 2). In agreement with the clustering analysis, the ex-neotype culture of *M. anisopliae s. str.* (CBS 130.71) clustered with the strains known as *M. lepidiotae*, forming a strongly supported monophyletic clade branching from a deep node at the base of the PARB and MGT clades (Fig. 2), supporting the view that *M. lepidiotae* should be synonymized with *M. anisopliae s. str*. Other strains designated as *M. anisopliae s. lat.* formed a separate strongly supported monophyletic clade (i.e. *M. neoanisopliae* sp. nov.) while ARSEF 549 (*M. hybridum* sp. nov.) branched at the base of *M. neoanisopliae* and was shown above to have a mixed ancestry. These two taxa are thus considered to be different species. Two strongly supported monophyletic clades were observed for the strains considered as *M. pingshaense s. lat.* (Fig. 3)*.* One clade contained the type strain of *M. pingshaense* (CBS 257.90) and so is considered *M. pingshaense s. str.* while the other clade is recognized here as a new species (*M. parapingshaense* sp. nov.).

The monophyletic clades supporting different species were also recovered in the coalescence-based species tree (Additional File 2: Fig. S1). The inference of the coalescent multi-species tree reconciliates the topologies of the 238 genes into the most probable species tree. This latter method corresponds to the phylogenetic species concepts based on the concordance between different gene trees (Taylor et al. 2000; Maharachchikumbura et al. 2021) and can outperform the concatenated genes-based approach (Liu et al. 2015). Yet, our results using this method confirm the species hypotheses formed with the concatenated-genes tree; all the clades supporting different species were supported with 100% posterior probability in the coalescent multispecies tree (Fig.2).

### Morphological analysis

**Fig. 4:**
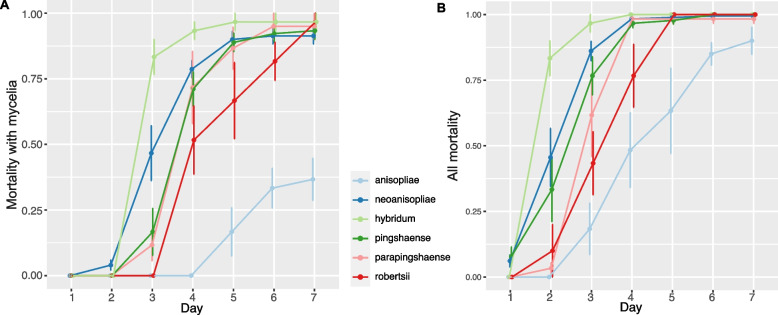
Virulence of *Metarhizium* species within the PARB group (*M. anisopliae sensu* *stricto*, *M. hybridum*, *M. neoanisopliae*, *M. parapingshaense*, *M. pingshaense*, *M. robertsii*) along 7 days of observation. **A** The mortality with mycelia; only dead insects covered by fungal mycelia and spores were counted. **B** All mortality; all dead insects including those manifesting no fungal material on the external surface were counted. The error bars represent the standard errors of respective data points

Based on the measurements from axenic cultures (PDA), we statistically analysed whether clades/strains considered to be distinct but closely related species possessed different morphological features in terms of the width and the length of the phialides and conidia (Fig. 3). We observed a significant difference in the width of conidia between *M. neoanisopliae* and *M. hybridum* (F = 31.323, *p*-value = 0.002) but this was non-significant for the other traits (Additional File 1: Table S2). For the *M. pingshaense s. lat.* group, there was a significant difference in the length of phialides in culture between *M. pingshaense* and *M. parapingshaense* (F = 6.421, p-value = 0.044) but not for the other traits (Additional File 1: Table S2). We also tested whether *M. anisopliae s. str.* would have different ranges of conidia and phialides width/length to the *M. anisopliae s. lat.* group (*M. hybridum* + *M. neoanisopliae*); surprisingly, none of the traits were found to be significantly different between these two groups (Additional File 1: Table S2). We found that these traits highly overlapped between different *Metarhizium* groups (Fig. 4). We also obtained measurements from a dry specimen each from *M. pingshaense* (MY5150) and *M. parapingshaense* (MY12337) (Additional File 1: Table S2). There was a significant difference in the width (F = 28.583, *p*-value = 8.676e-07) and the length (F = 96.174, *p*-value = 2.968e-15) of conidia between these two strains, each representating different species.

### Virulence

Our statistical analysis revealed a significant difference in both the unconditional mortality (UM) and the mortality with mycelia (MM) between the species within the PARB group (UM: F = 11.140, *p*-value < 0.0001; MM: F = 27.078, *p*-value < 0.0001) as well as between the days of observation (UM: F = 152.054, *p*-value < 0.0001; MM: F = 169.690, *p*-value < 0.0001) (Fig. 4). Considering the pairwise difference between species, *M. anisopliae s. str.* were almost always significantly less virulent than all species within the PARB group for both types of mortality (Additional File 1: Table S3). There was no significant difference between neither *M. parapingshaense* and *M. pingshaense* (UM: t = -1.588, *p*-value = 0.567; MM: t = -0.070, *p*-value = 1.000), nor *M. neoanisopliae* and *M. hybridum* (UM: t = 1.148, *p*-value = 0.755; MM: t = -1.708, *p*-value = 0.528), respectively. Interestingly, a notable variation of mortality between strains within species was observed (Additional File 3: Fig. S2).

### Metabolomics

**Fig. 5:**
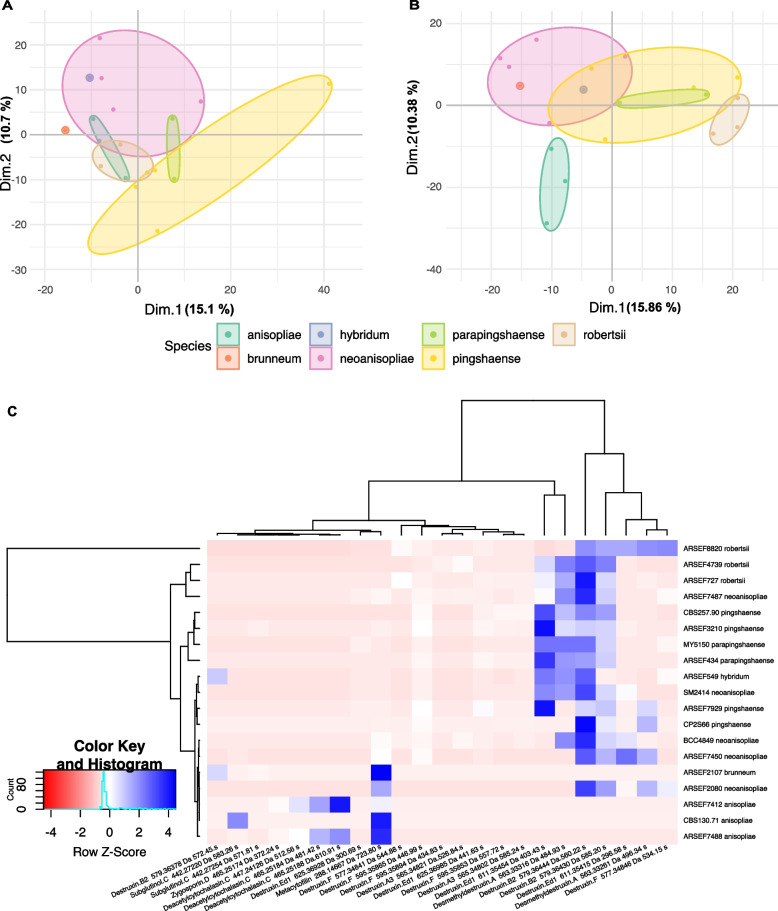
Metabolomic analyses based on peak area data obtained from liquid chromatography-mass spectrometry (LC–MS) for *Metarhizium* species of the PARB group. **A** Principal component analysis (PCA) of data from cell extracts. **B** PCA of data from broth extracts. The ellipsoids delimit the perimeter of the sample distributions. **C** A heatmap based on Manhattan distance clustering for annotated compounds.

The PCA on the data of peak area of selected features showed that, for both the broth extract (BE) and cell extract (CE), most of the samples had the distribution of secondary metabolites overlapped without any clear difference between species (Additional File 4: Figure S3). When categorizing different taxa into species complexes based on the phylogenomics results: **PARB**–*M. brunneum*, *M. hybridum*, *M. neoanisopliae*, *M. parapingshaense*, and *M. pingshaense*; **Anisopliae Strict** (*M. anisopliae s. str.*); **MGT**–*M. majus, M. guizhouense, M. clavatum, M. gryllidicola, M. kalasinense, M. phasmatodea,* and *M. sulphureum*; **Acridum**–*M. acridum* and *M. globosum*; **Flavoviride**–*M. flavoviride* and *M. frigidum*; no discriminating pattern could be observed between these species complexes (Additional File 5: Figure S4). However, by narrowing the data to only the PARB group, in which we propose three new species and *M. anisopliae s. str.*, we could observe for CE a segregation, to an extent, between *M. pingshaense* and *M. neoanisopliae*. However, *M. parapingshaense* and *M. neoanisopliae* largely overlapped respectively with *M. pingshaense* and *M. anisopliae s. str.* (Fig. 5A). Interestingly, for BE, *M. neoanisopliae* clearly segregated from *M. anisopliae s. str.* while *M. parapingshaense* and *M. pingshaense* were found to overlap largely between them (Fig. 5B).

**Fig. 6:**
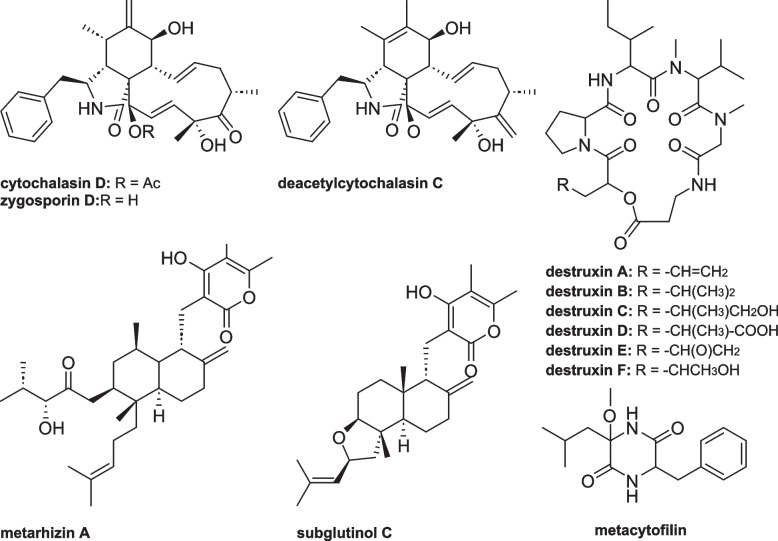
Representative secondary metabolites found in species of the *Metarhizium anisopliae* species complex including cytochalasins, destruxins, metarhizins, subglutinols, and metaytofilins

Most of the identified known compounds produced by our fungal strains belong to various secondary metabolite families such as cytochalasins, destruxins, metarhizins, subglutinols, and metacytofilins (Fig. 6, Additional File 6). By focusing on the annotated compounds between PARB and *M. anisopliae s. str.*, the Manhattan distance-based heatmap showed that *M. anisopliae s. str.* tended to closely group, and produce to a lesser extent various destruxins compared to the strains from the PARB group (Fig. 5C).

## Taxonomy

***Metarhizium anisopliae*** (Metsch.) Sorokīn, *Plant Paras. Man Anim*. **2**: 268 (1883).

(Fig. 7).

**Fig. 7:**
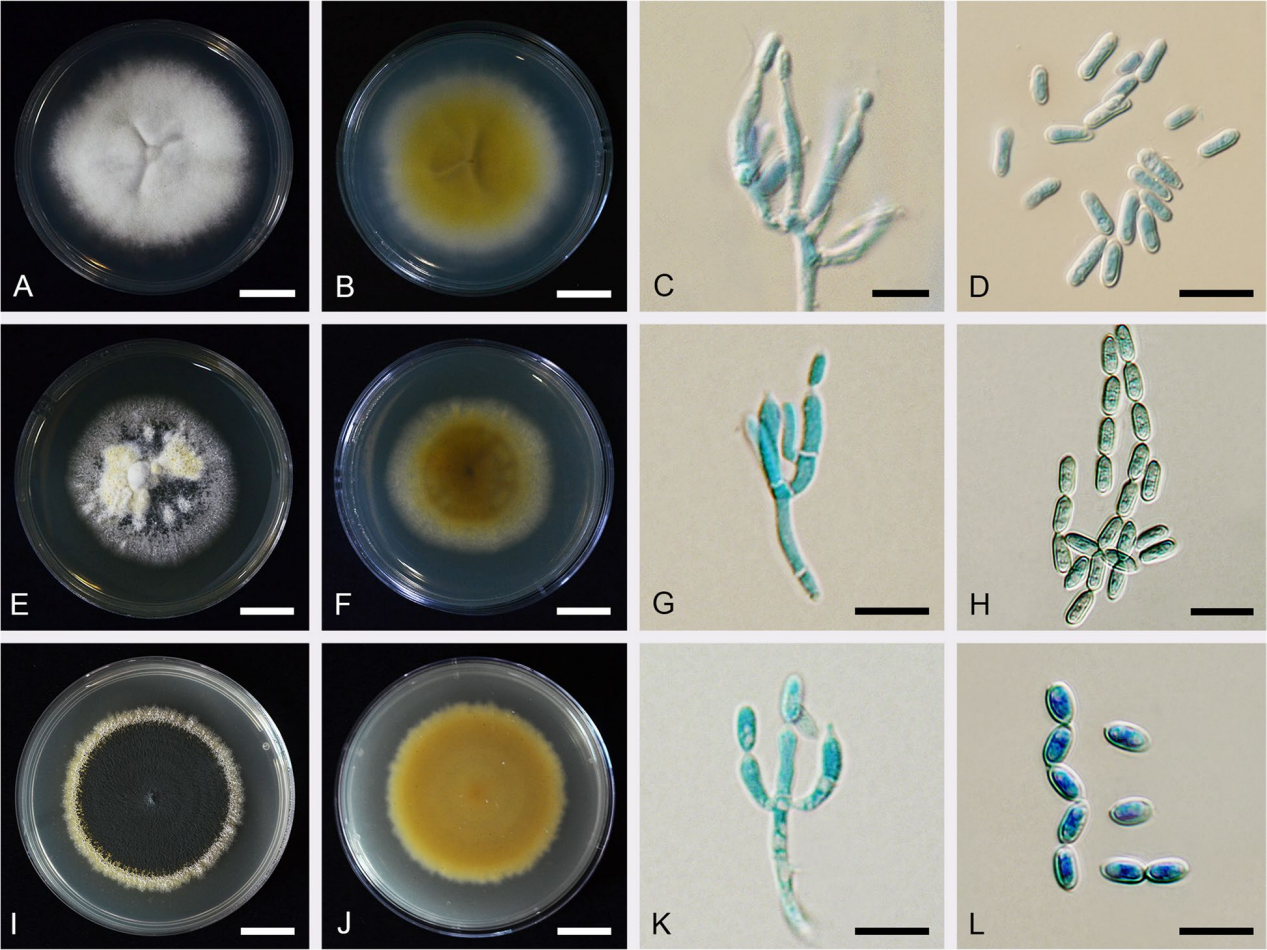
*Metarhizium anisopliae *sensu stricto: CBS 130.71, **A** colony obverse on PDA after 2 wk. **B** colony reverse on PDA after 2 wk. **C** Phialides and conidia on PDA. (**D**) conidia on PDA.; ARSEF 7412, **E** colony obverse on PDA after 2 wk. **F** colony reverse on PDA after 2 wk. **G** Phialides and conidia on PDA. **H** conidia on PDA.; ARSEF 7488 (*M. lepidiotae* culture ex-type), **I** colony obverse on PDA after 2 wk. **J** colony reverse on PDA after 2 wk. **K** Phialides and conidia on PDA. **L** conidia on PDA. — Scale bars: b − c = 10 mm; d − f = 10 µm

*Basionym*: *Entomophthora anisopliae* Metsch., *Zap. Imp. Obshch. Khoz. Ross.*: 45 (1879).

*Synonyms*: *Isaria anisopliae* (Metsch.) Pettit, *Cornell Univ. Agric. Exp. St. Bull.* **97**: 356 (1895).

*Penicillium anisopliae* (Metsch.) Vuill., *Bull. Trimest. Soc. Mycol. Fr*. **20**: 221(1904).

*Isaria destructor* Metsch., *Zool. Anz.* **3**: 45 (1880).

*Oospora destructor* (Metsch.) Delacroix, *Bull. Trimest. Soc. Mycol. Fr.* **9**: 260 (1893).

*Isaria anisopliae* var. *americana* Pettit, *Cornell Univ. Agric. Exp. St. Bull.* **97**: 354 (1895).

*Penicillium cicadinum* Höhn., *Sber. Akad. Wiss. Wien* **118**: 405 (1909).

*Metarhizium cicadinum* (Höhn.) Petch, *Trans. Brit. Mycol. Soc*. **16**: 68 (1931).

*Myrothecium commune* Pidopl. & Kiril., *Mikrobiol. Zh.* **31**: 159 (1969).

*Sporotrichum paranense* Marchionatto, *Bol. Mens. Min. Agric. Noac. Buenos Aires* **34**: 241 (1933).

*Metarhizium anisopliae* var. *lepidiotae* Driver & Milner, *Mycol. Res.* **104**: 145 (2000); as “*lepidiotum*”.

*Metarhizium lepidiotae* (Driver & Milner) J.F. Bisch. et al*., Mycologia* **101**: 520 (2009).

*Type*: **Ukraine**, isolated from *Avena sativa* root, collection date unknown, *A.A. Milko* (CBS H-14432 – neotype of *Metarhizium anisopliae* preserved in a metabolically inactive state; cultures ex-neotype CBS 130.71 = ATCC 22269 = VKM F-1490).

*Habitat:* Various insect hosts, soil.

*Distribution:* Worldwide.

*Description: Colonies* on PDA attaining a diameter of 4–4.5 mm after 14 d, white, grey green (N189), flat, entire edge, pale yellow border, grey green of colonies due to production of conidia. Sporulation starts at 7 d after inoculation, reverse greenish yellow (153B), pale yellow in the margin of the colony. *Conidiophores* terminating in branches with 2–3 phialides per branch. *Phialides* cylindrical with semi-papillate apices, (5–)7–13.5(–25) × (1.5–)2–3 µm. *Conidia* smooth-walled, cylindrical with round apices, (5–)6–9.5(–14.5) × (2–)3–4(–5) µm.

*Notes*: Our phylogenomic analyses indicated that the ex-type strain *M. lepidiotae* ARSEF 7488, and ARSEF 7412, cluster with the ex-neotype *M. anisopliae* CBS 130.71 from Ukraine. Therefore, this species is synonymized with *M. anisopliae s. str*. The samples of *M. anisopliae s. str.* showed greater variability in conidial length and width than *M. neoanisopliae* sp. nov. and *M. hybridum* sp. nov., with a significant portion of conidia being longer (> 10 µm) and larger (> 3.5 µm) than *M. hybridum* and *M. neoanisopliae*. The virulence of *M. anisopliae* against *Spodoptera exigua* (*Lepidoptera*) is less than other species of PARB Clade (*M. hybridum*, *M. neoanisopliae*, *M. parapingshaense*, *M. pingshaense*, and*M. robertsii*).

***Metarhizium hybridum*** Kobmoo, Mongkolsamrit & Khonsanit, **sp. nov.**

**(**Fig. 8).

**Fig. 8:**
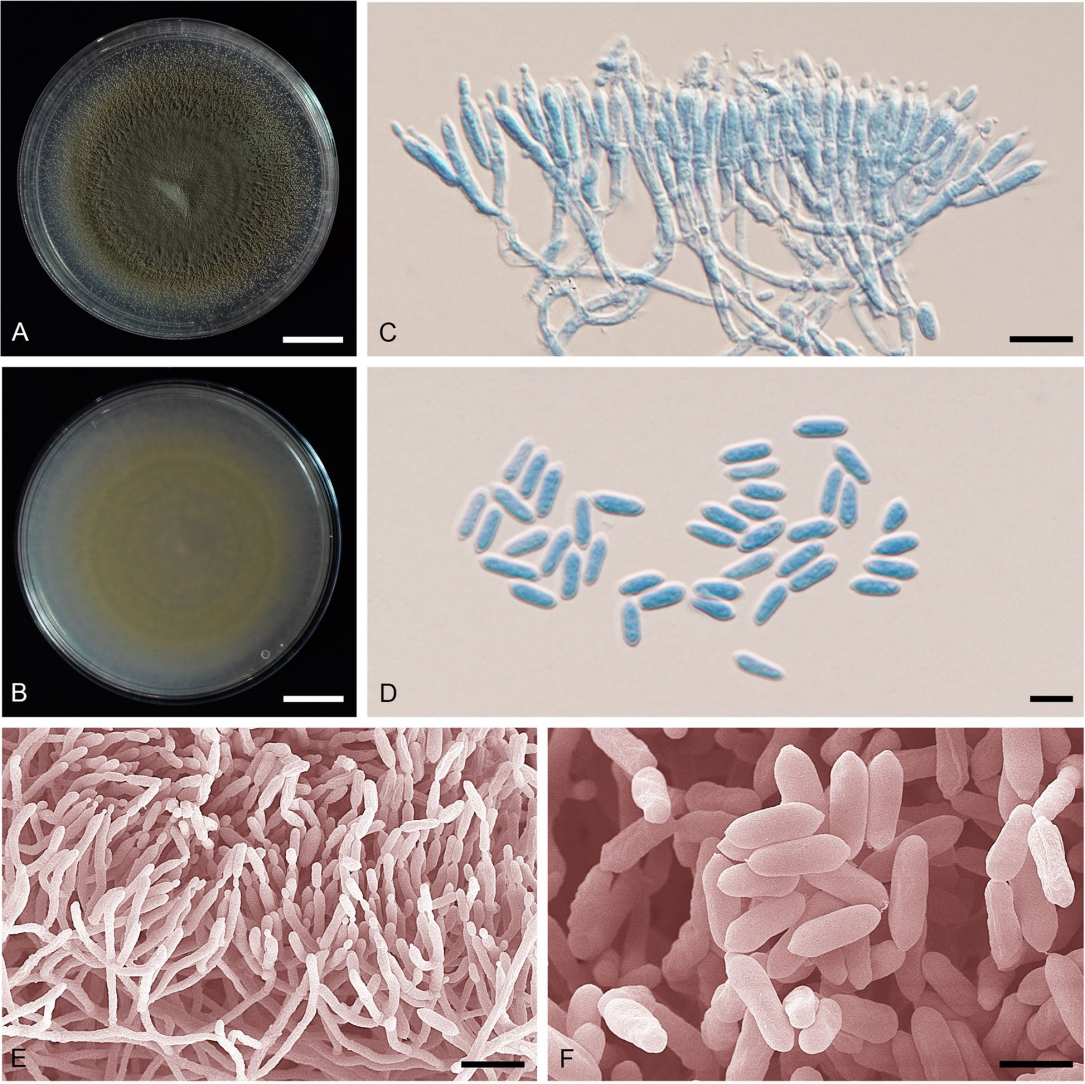
*Metarhizium hybridum* (ARSEF 549). **A** Colony obverse on PDA after 2 wk. **B** Colony reverse on PDA after 2 wk. **C** Phialides and conidia on PDA. **D** Conidia on PDA. **E** Scanning electron micrographs of phialide with conidia on PDA. **F** Scanning electron micrographs of conidia on PDA. — Scale bars: a − b = 10 mm; c, e = 10 µm; d, f = 5 µm

MycoBank MB 850069.

*Etymology:* The species name is derived from the genetic mixed ancestry inferred from genomic analyses.

*Diagnosis*: *Metarhizium hybridum* is highly similar to its sister species *M. neoanisopliae* sp. nov., but has conidial width statistically smaller than the latter. The conidial width is always less than 3.5 µm, and the conidial length, like its sister species *M. neoanisopliae*, is always shorter than 8.5 µm (reserved for *M. anisopliae*). It is also more virulent against *Spodoptera exigua* than *M. anisopliae s. str*.

*Type:* **Brazil**, isolated from unknown source, collection date unknown, *D.W. Robert*, (BBH50656 – holotype preserved in a metabolically inactive state; ARSEF 549 – culture ex-type).

*Description*: Colonies on PDA medium attaining 45–48 mm diam after 14 d, flat, entire edge, white to pale yellow, turning moderate yellow-green (148A). Sporulation starts at 5 d after inoculation, reverse light yellow (160B). *Conidiophores* terminating in branches with 2–3 phialides per branch. *Phialides* cylindrical with semi-papillate apices, (5–)6–10.5(–12) × 2–2.5 µm. *Conidia* smooth-walled, ellipsoidal to cylindrical with round apices, (5–)6–8 × 2–3.

*Distribution*: Brazil.

*Notes*: The genomic data showed this species to have a mixed ancestry, which is distinct from that of *M. neoanisopliae* and *M. anisopliae s. str*.

***Metarhizium neoanisopliae*** Kobmoo, Mongkolsamrit, Noisripoom & Khonsanit, **sp. nov.**

(Fig. 9).

**Fig. 9:**
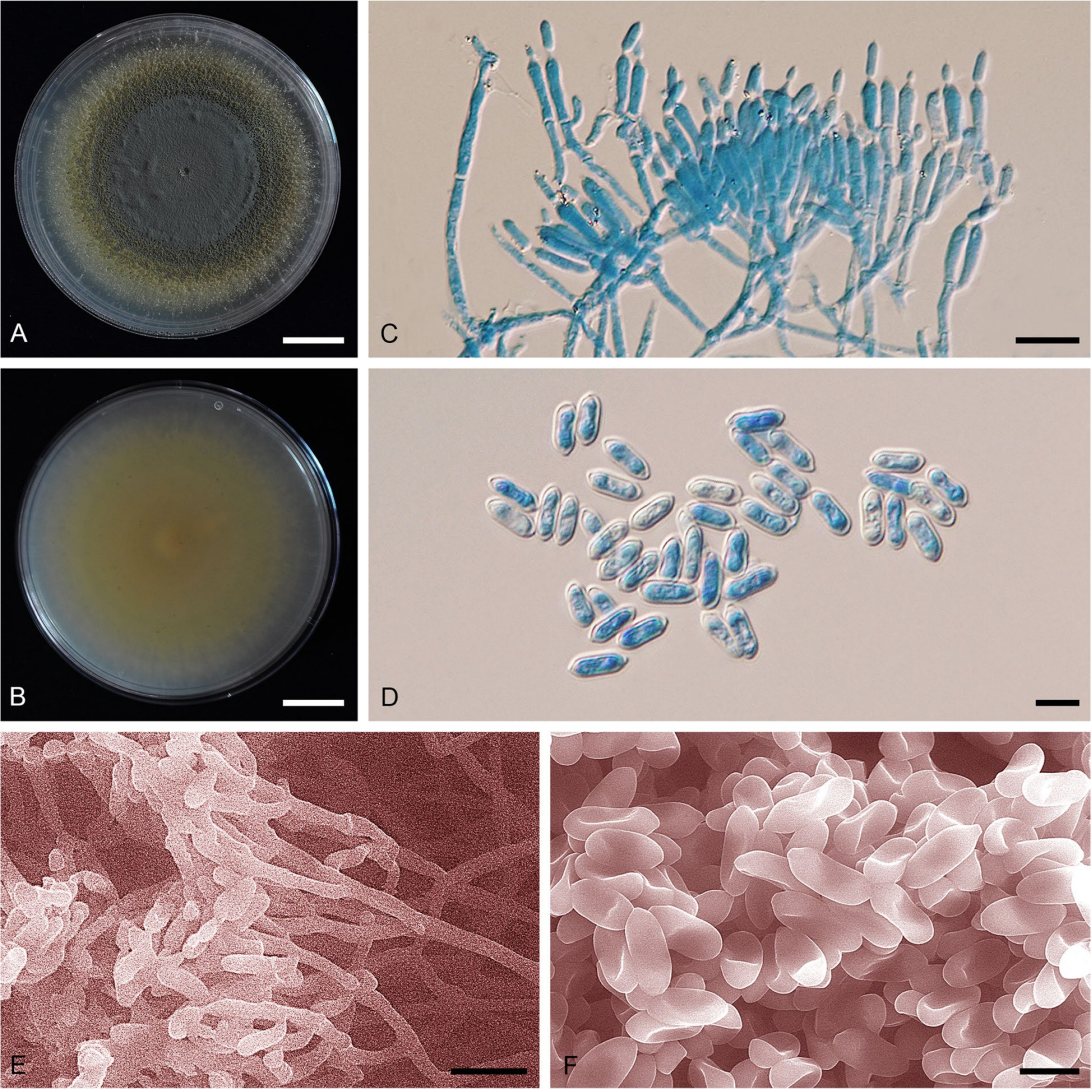
*Metarhizium neoanisopliae* (ARSEF 7487). **A** Colony obverse on PDA after 2 wk. **B** Colony reverse on PDA after 2 wk. **C** Phialides and conidia on PDA. **D** Conidia on PDA. **E** Scanning electron micrographs of phialide with conidia on PDA. **F** Scanning electron micrographs of conidia on PDA. — Scale bars: a − b = 10 mm; c, e = 10 µm; d, f = 5 µm

MycoBank MB 850088.

*Etymology:* Morphologically resembling *Metarhizium anisopliae* but phylogenetically distinct.

*Diagnosis*: *Metarhizium neoanisopliae* is similar to its sister species *M. hybridum* sp. nov. but the conidial width is statistically larger than the latter. The conidial width is never below 2 µm, and can be beyond 3.5 µm, but never surpasses 4 µm as in *M. anisopliae*. The conidial length, as in its sister species *M. hybridum*, is never longer than 8.5 µm. It is more virulent against *Spodoptera exigua* than *M. anisopliae s. str*.

*Type:* **Eritrea**, isolated from *Schistocerca gregaria* (*Orthoptera*), 2 Apr 1965, *K.H. Veen* (CBS H-7330 – holotype preserved in a metabolically inactive state; cultures ex-type CBS 289.67 = IMI 168777; derivative cultures ARSEF 7487 = IMI 168777ii derived from locust experimentally infected with IMI 168777).

*Habitat/host*: *Coleoptera, Delphacidae, Hemiptera, Orthoptera*, soil, wood.

*Description*: Colonies on PDA medium attaining 45–48 mm diam after 14 d, flat, entire edge, white to pale yellow, turning moderate yellow-green (147C) to greyish olive green (NN137C-D). Sporulation starts at 5 d after inoculation, reverse pale yellow (162D). *Conidiophores* terminating in branches with 2–3 phialides per branch. *Phialides* cylindrical with semi-papillate apices, (5–)7.6–10(–15) × 2–3 µm. *Conidia* smooth-walled, ellipsoidal to cylindrical with round apices, (6–)6.5–7.5(–8) × 2.5–3(–3.5) µm.

*Additional material examined*: **Thailand**: Chanthaburi Province, Chanaphon Mangosteen Orchard, soil, 16 Nov 2021, *S. Mongkolsamrit*; *U. Pinruan* (BCC 96565, BCC 96571, BCC 96576, BCC 96578, BCC 96580, BCC 96581, BCC 96583); Chiang Mai, Doi Inthanon National Park, 28 Sep 1998, *W. Kladwang* (BCC 4849). **Indonesia**: *Hemiptera* (*Delphacidae*, *Nilaparvata lugens*), 1985, *H. Soeharto* (ARSEF 2080). **Australia**: Queensland, *Coleoptera* larva (*Scarabaeidae*, *Heteronyx piceus*), 11 Jun 1992, collector unknown (ARSEF 7450).

***Metarhizium parapingshaense*** Kobmoo, Mongkolsamrit & Khonsanit, **sp. nov.**

**(**Fig. 10).

**Fig. 10:**
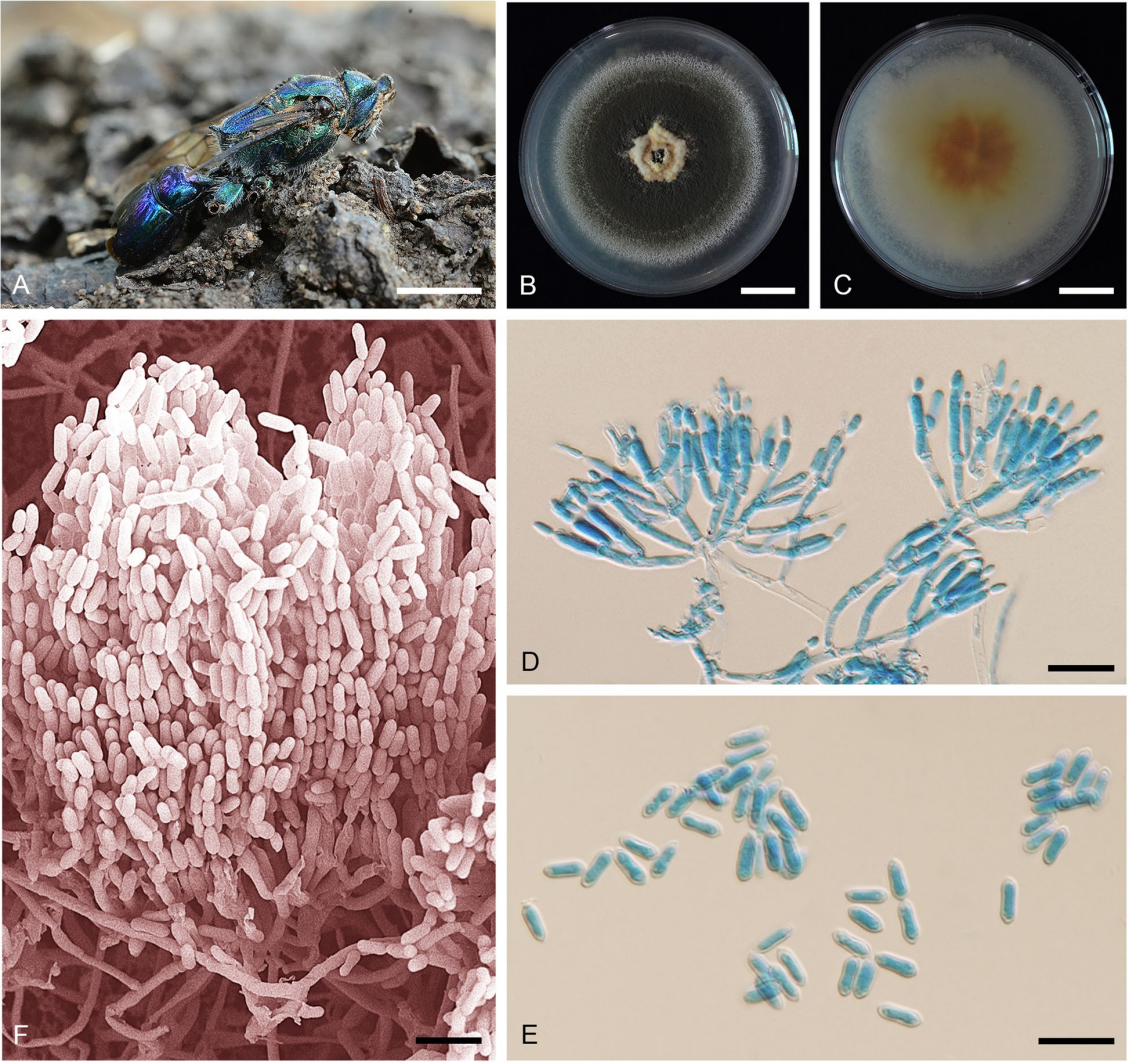
*Metarhizium parapingshaense* (MY 5150, BCC 37941). **A** Fungus on an adult wasp (*Hymenoptera*). **B** Colony obverse on PDA after 2 wk. **C** Colony reverse on PDA after 2 wk. **D** Phialides and conidia on PDA. **E** Conidia on PDA. **F** Scanning electron micrographs of phialide with conidia on PDA. — Scale bars: b − c = 10 mm; d − f = 10 µm

MycoBank MB 850087.

*Etymology:* The epithet refers to the very close phylogenetic position to *Metarhizium pingshaense*, a sister species evolving in parallel from a common ancestor.

*Diagnosis*: *Metarhizium parapingshaense* is very similar to its sister species, *M. pingshaense,* but differs in conidial size. The conidia on specimens are statistically larger for *M.* *parapingshaense* (mostly > 6 µm in length and frequently > 2.5 µm in width). The phialides produced on PDA are also statistically shorter than in *M. pingshaense*, with the phialide length never over 12.5 µm (as occurs in the latter).

*Type*: **Thailand**: *Nakhon Ratchasima Province*: Khao Yai National Park, isolated from a wasp (*Hymenoptera*) on leaf litter, 16 Aug 2009, *K. Tasanathai, P. Srikitikulchai, S. Mongkolsamrit, T. Chohmee & R. Ridkaew* (BBH 26560 – holotype preserved in a metabolically inactive state; BCC 37941 – ex-type culture).

*Description*: Colonies on PDA medium attaining 40–42 mm diam after 14 d, dark green, flat, entire edge, border white, dark green of colonies due to production of conidia, pale yellow in the middle of the colony. Sporulation starting 5 d after inoculation, reverse pale yellow (162D), orange in the middle of the colony. *Conidiophores* terminating in branches with 2–3 phialides per branch. *Phialides* cylindrical with semi-papillate apices, (5–)6–10(–12) × (1.5–)2–2.5(–3) µm. *Conidia* smooth-walled, cylindrical with round apices, (7–)7.5–9(–10) × 2–2.5(–3) µm.

*Habitat/host*: *Coleoptera*, *Hymenoptera*, soil.

*Distribution*: Solomon Islands, Thailand.

*Notes*: *Metarhizium parapingshaense* is separated from its sister species *M. pingshaense* based on genetic segregation, following the phylogenetic species concept, from genome-wide polymorphisms. The conidia on specimens are also statistically larger and longer than the latter.

*Additional material examined*: **Thailand**: *Chanthaburi Province*: Chanaphon Mangosteen Orchard, soil, 16 Nov 2021, *S. Mongkolsamrit & U. Pinruan* (BCC 96582).—**Solomon Islands**: *Coleoptera* larva, 6 Jun 1994, *W. Theunis* (ARSEF 4342).

## Discussion

### Hidden Diversity within the *Metarhizium anisopliae* species complex

Our population genomics-based phylogenetic results agree with previous taxonomic treatments of the genus *Metarhizium *(Bischoff et al. 2009; Kepler et al. 2014; Mongkolsamrit et al. 2020) in the sense that the *M. flavoviride* complex is a sister clade to the *M. anisopliae* species complex and that, within the *M. anisopliae* complex, *M. acridum* and *M. globosum* form a sister clade to the rest of the complex. We recognized three new species within the *M. anisopliae* complex, namely *M. neoanisopliae*, *M. hybridum,* and *M. parapingshaense*. *Metarhizium neoanisopliae* and *M. hybridum* are distinguished from *M. anisopliae s. str.* based on multiple lines of evidence. First, the genomics data segregated *M. neoanisopliae* and *M. hybridum* from *M. anisopliae s. str.* Second, the virulence data showed *M. anisopliae s. str.* to be less virulent than *M. neoanisopliae* and *M. hybridum* in laboratory conditions. Finally, the metabolomics analysis also revealed the differences in secondary metabolite production between the two former taxa and the latter. These support the distinct species status of *M. neoanisopliae* and *M. hybridum* from *M. anisopliae s. str.*

The original description with illustration of *M. anisopliae *by Metchnikoff (1879) is in Russian, and currently not accessible. The oldest account of the morphology of this species, that we could find, was that of Delacroix (1893), who stated that he had examined specimens of Metchnikoff and described the length of *M. anisopliae*’s conidia as 7–15 µm. Veen (1968) and Tulloch (1976) made a reference to Metchnikoff’s description of *M. anisopliae* as having conidia of 4.8 µm long and 1.6 µm wide. The examination of putative *M. anisopliae *strains by Veen (1968) and Tulloch (1976) resulted in the conidia length being respectively at 4.6–11.5 µm and 3.5–9 µm. These measurements are smaller than those of Delacroix (1893) and of *M. anisopliae s. str.* as interpreted in our study, but more within the range of *M. neoanisopliae*. Overall, the conidial dimension of *M. anisopliae s. str. *with CBS 170.71 as the neotype (Mongkolsamrit et al. 2020) better fits the account of Delacroix (1893). Taken with the fact that CBS 170.71 came from the same original locality of Metchnikoff’s species, it is justifiable to accept this isolate as the neotype of *M. anisopliae s. str. *(Mongkolsamrit et al. 2020). This reclassification means that ARSEF 7450 and many strains previously identified as *M. anisopliae* should be reidentified as *M. neoanisopliae*.

With CBS 170.71 accepted as the ex-neotype culture of *M. anisopliae s. str.*, *M. lepidiotae* should be considered an objective synonym of *M. anisopliae s. str.* This proposition is supported by the clustering of the ex-type strain of *M. lepidiotae* (ARSEF 7488) with CBS 170.71. Initially described as a variety of *M. anisopliae *by Driver et al. (2000), *M. lepidiotae *was later elevated to species rank by Bischoff et al. (2009), primarily based on molecular phylogeny. Notably, the spore dimensions of *M. lepidiotae *(conidia 7.3–10.6 × 3–4.1 µm; Driver et al. 2000) falls within the range of *M. anisopliae s. str.*

The difference between *M. neoanisopliae* and *M. hybridum* principally relies on genomic data. As *M. hybridum* clustered next to *M. neoanisopliae* and was represented by a single strain, one might argue that *M. hybridum* should be considered as *M. neoanisopliae*. However, *M. hybridum* clearly demonstrated a genomic signature of mixed ancestry, which is not the case for *M. neoanisopliae*. Furthermore, the conidial width of these two species is significantly different, those of *M. hybridum* being slightly narrower than those of *M. neoanisopliae*. Although this difference is not easily perceptible by eye, it is statistically significant.

The distinction between *M. parapingshaense* and *M. pingshaense* is supported by the genomic data. This showed them to form closely related but distinct clades. In addition, morphological data show that the former has statistically larger on-specimen conidia than the latter, although some individual spores have an overlapping size. The virulence between these two species is at the same level and, like *M. neoanisopliae* and *M. hybridum*, higher than that of *M. anisopliae*. Overall, it is well supported that *M. anisopliae sensu* *stricto* is different to the novel species proposed in this study.

### Evolution of virulence and host specificity of the *Metarhizium anisopliae* complex

The phylogeny of the *M. anisopliae* complex shows that *M. acridum* and *M. globosum* formed a sister clade to the other species. *Metarhizium acridum* is well documented as a pathogen specific to *Orthoptera *(Wang and Leger 2005; Hu et al. 2014) while the other species are recognised as intermediate (MGT group sensu Bischoff: *M. guizhouense, M. majus*) to the broad-range generalist (PARB group sensu Bischoff: *M. anisopliae*, *M. brunneum*, *M. pingshaense,* and *M. robertsii*) pathogens (Bischoff et al. 2009; Hu et al. 2014). The close position of *M. acridum* to the most recent common ancestor (MRCA) of the *M. anisopliae *complex have led some authors to formulate the generalization that it “evolved from specialists” via “transitional species with intermediate host ranges” (Hu et al. 2014). Without going into the controversy of whether such a statement is accurate, we can accept that the *M. anisopliae* complex comprises two distinct groups, one formed by *M. acridum* and *M. globosum* which are specialists, and the other consisting of ones with intermediate to very broad host ranges. Therefore, there are distinct evolutionary trajectories into different levels of host specificity. This situation is in contrast with the genus *Beauveria*, in which most of the species were described as broad generalists; many strains, including the types, were found associated with *Coleoptera*, the most species-rich order of insects, with some species appearing to become more specialized on other arthropod groups such as *Orthoptera* (*B. gryllidicola*, *B. namnaoensis*), *Lepidoptera* (*B. pseudobassiana*, *B. thailandica*) or *Acari* (*B. varroae*) (Kobmoo et al. 2022). The specialization to other insects besides *Coleoptera* appeared independently multiple times in that genus.

As our study only focused on the virulence of *Metarhizium* spp. towards an insect (*Spodoptera exigua*), this does not have the power to refute any hypothesis regarding host specificity, but it did show that the variation of virulence could be partly explained by evolutionary history. *Metarhizium anisopliae s. str.* (syn. *M. lepidiotae*), which phylogenetically branched directly from the MRCA of the *M. anisopliae* complex, is clearly less virulent than other species considered to be *M. anisopliae s. lat.* (PARB group; i.e. *M. hybridum*, *M. neoanisopliae*, *M. parapingshaense*, *M. pingshaense,* and *M. robertsii*). It is tempting to hypothesize that the *M. anisopliae* complex evolved from a moderately entomopathogenic common ancestor to become highly entomopathogenic. The difference of virulence between *M. anisopliae s. str.* and other species of the complex has an implication in the development of biocontrol strategies as many of the *Metarhizium* strains used in biocontrol have been putatively identified as *M. anisopliae *based solely on ITS (Dong et al. 2016; Alam 2019; Ahmed et al. 2020; Qubbaj and Samara 2022) while they might actually represent different species with different virulence patterns. The difference in virulence is probably explained by the PARB group species which produce more specific destruxins and demethyldestruxins as shown by our metabolomic analyses. Destruxins are well known for their insecticidal activity (Pedras et al. 2002; Wang et al. 2004; Pal et al. 2007). Our data warrant further research on specific cyclic peptides produced by highly virulent strains.

*Metarhizium *is known to be ecologically versatile (St. Leger and Wang 2020), capable of being soil-borne, associated to plant rhizospheres (Vega et al. 2009), and even occasionally pathogenic to vertebrates (Horgan et al. 2022). Entomopathogenicity can be viewed as an ecologically specialized function within the genus. A lesser entomopathogenic MRCA of the *M. anisopliae* complex would support this hypothesis. However, it is necessary to gain more virulence data across a larger panel of strains from the *M. anisopliae* complex (e.g. MGT group) in order to accurately construct an actual ancestral reconstruction.

### Persistence of a biocontrol candidate strain in field application

Some strains of *M. neoanisopliae* were isolated from the soil of a fruit orchard where BCC 4849 had been applied as biocontrol agent against insect pests two years earlier. Some of these strains were inferred to be of the same clonal lineage as BCC 4849. This shows that this candidate strain can persist in the field for several years. Similarly, a few studies have showcased the persistence of biocontrol strains of *Beauveria *(Mei et al. 2020) and *Metarhizium *(Peng et al. 2021) in the field many years after their initial applications. These findings, with ours, are encouraging as they show that biocontrol strains could be sustainably used in long-term efforts to reduce the utilization of chemical pesticides and ensure better food security.

## Conclusion

We combined multiple lines of evidence in resolving the taxonomy of a species complex. Fungal taxonomy in the twenty-first century has increasingly relied mostly on molecular data and the phylogenetic species concept (Taylor et al. 2000). Particularly for *Ascomycota*, molecular phylogenetics provides the basis to delineate evolutionary lineages that frequently do not match other characteristics (Dugan and Everhart 2018; Balasundaram et al. 2015; Leavitt et al. 2016; Mongkolsamrit et al. 2018; Kobmoo et al. 2021; Steenwyk 2023). We have demonstrated here that evolutionary lineages based on whole-genome sequencing can be used to seek for differences in other aspects of fungal biology. The metabolomics and virulence data confirmed the difference between *M. anisopliae s. str.* and the rest of the species complex while the morphometrics analysis revealed fine-scale difference between closely related species. This work has also taken advantage of isolates stored in various institutional collections, allowing the clarification of the phylogenetic positions of ex-type cultures, and demonstrated the importance of integrating a vast panel of specimens across different institutions. Institutional collections are rich sources of undiscovered or misidentified species that can only be uncovered using multiple lines of evidence (molecular, genomics, metabolomics, ecology). Such integrative approach based on inter-disciplinary and inter-institutional collaborations can greatly benefit future taxonomic work of fungi.

## Supplementary Information

Additional file 1. Supplementary Tables

Additional file 2: Figure S1. A coalescent-based species tree based on the reconciliation of 238 gene trees. The nodes are shown with values of posterior probability

Additional file 3: Figure S2. Intraspecific variation of virulence. Mortality with mycelia: only dead insects covered by fungal mycelia were counted.Unconditional mortality: all dead insects were counted. The error bars represent standard errors

Additional file 4: Figure S3. Metabolomic analyses based on peak area data obtained from liquid chromatography-mass spectrometryfor all *Metarhizium* species included in this study. Principal component analyses of data from cell extracts, and broth extracts.

Additional file 5: Figure S4. Metabolomic analyses based on peak area data obtained from liquid chromatography-mass spectrometry: comparison between different species complexes. Principal component analyses of data from cell extracts, and broth extracts. Acridum = *M. acridum* complex [*M. acridum* + *M. globosum*]; Anisopliae strict = *M. anisopliae sensu stricto*; Flavoviride = *M. flavoviride* complex [*M. flavoviride* + *M. frigidum*]; MGT = MGT group [*M. majus, M. guizhouense, M. clavatum, M. gryllidicola, M. kalasinense, M. phasmatodea* and *M. sulphureum*]; PARB = PARB group [*M. brunneum*, *M. hybridum*, *M. neoanisopliae*,*M. parapingshaense*, *M. pingshaense*].

Additional file 6. Peak area data of annotated compounds from LC-MS

## Data Availability

The raw sequencing reads generated in this study were deposited at the NCBI Sequence Reads Archive (SRA) (PRJNA1111679). The single nucleotide polymorphisms data under the VCF format is available at Dryad data repository (10.5061/dryad.q83bk3jr8). The fungal type materials and type strains of the new species proposed in this study were deposited at BIOTEC Bangkok Herbarium (BBH) and BIOTEC Culture Collections (BCC) with the accession numbers provided in Table 1.

## Abbreviations

ARSEF: Agricultural Research Service Collection of Entomopathogenic Fungal Cultures
BBH: BIOTEC Bangkok Herbarium
BE: Broth extract
BCC: BIOTEC Culture Collection
BIOTEC: National Center for Genetic Engineering and Biotechnology
CBS: Centraal Bureau voor Schimmelcultures, Baarn, The Netherlands (Central Bureau of Fungal Cultures)
KNAW: Koninklijke Nederlandse Akademie van Wetenschappen (Royal Dutch Academy of Sciences)
CE: Cell extract
Df (or df): Degree of freedom
F84: Felsenstein 84 distance
IMI: International Mycological Institute (now part of Commonwealth Agricultural Bureau International or CABI)
LC-MS: Liquid chromatography-Mass spectrometry
ML: Maximum likelihood
NSTDA: National Science and Technology Development Agency
PCA: Principal Component Analysis
s. lat.: sensu lato
s. str.: sensu stricto
USDA: United States Department of Agriculture
WGS: Whole genome sequencing
wk: week
WI: Westerdijk Fungal Biodiversity Institute

## Declarations

### Adherence to national and international regulations

All the fungal strains used in this study have been legally obtained, respecting the Convention on Biological Diversity (Rio Convention). The experiments on insects were conducted following the regulation of the Institute of Animals for Scientific Purposes Development (IAD), National Research Council of Thailand (NRCT) with Dr. Noppol Kobmoo having a permit for conducting animal research.
