# Supplementary material for: Integrative taxonomy of Metarhizium anisopliae species complex, based on phylogenomics combined with morphometrics, metabolomics, and virulence data
Source: IMA Fungus. 2024 Sep 11;15:30. doi: 10.1186/s43008-024-00154-9 (PMC11389511; doi:10.1186/s43008-024-00154-9)
Supplement: Supplementary file 1 — Additional file 1. Supplementary Tables [file 43008_2024_154_MOESM1_ESM.pdf]

Supplementary Tables.

Table S1. Multi-locus clonal groups (MLG) inferred from genome-wide 98,085 single nucleotide polymorphisms.

MLG	Samples	Species
1	MY7440	*M. kalasinense*
2	MY7343	*M. kalasinense*
3	CBS258.90	*M. ghizhouense*
4	ARSEF6238	*M. ghizhouense*
5	MY4542	*M. sulphureum*
6	MY4504	*M. sulphureum*
7	MY5321	*M. sulphureum*
8	MY4561	*M. sulphureum*
9	MY4541	*M. sulphureum*
10	MY4549	*M. sulphureum*
11	MY4543	*M. sulphureum*
12	MY4552	*M. sulphureum*
13	MY4548	*M. sulphureum*
14	MY4547	*M. sulphureum*
15	MY4581	*M. sulphureum*
16	MY4545	*M. sulphureum*
17	NHJ11819	*M. sulphureum*
18	MY4546	*M. sulphureum*
19	MY4376	*M. sulphureum*
20	MY6023	*M. sulphureum*
21	NHJ14124	*M. sulphureum*
23	CP1-S38.1, PGP247.1	*M. neoanisopliae*
24	BCC4849, CP1-S24.1 (SM2387), CP2-S100 (SM2402), CP2-S77.1 (SM2398), CP2-S99.1 (SM2400)	*M. neoanisopliae*
25	ARSEF7487	*M. neoanisopliae*
26	ARSEF7450	*M. neoanisopliae*
27	ARSEF2080	*M. neoanisopliae*
28	PGP239.1	*M. neoanisopliae*
29	MY12337	*M. pingshaense*
30	CBS257.90	*M. pingshaense*
33	CP1-S36.1 (SM2931), CP2-S10 (SM2395), CP2-S66 (SM2397)	*M. pingshaense*

38	ARSEF3210	*M. pingshaense*
39	ARSEF7929	*M. pingshaense*
34	PGP252.1 (SM2415)	*M. parapingshaense*
35	MY5150	*M. parapingshaense*
36	ARSEF4342	*M. parapingshaense*
37	ARSEF549	*M. hybridum*
40	ARSEF2107	*M. brunneum*
41	ARSEF2596	*M. globosum*
47	MY11578, MY7483, NHJ11527, MY5085, MY3226, MY5073, MY5073, MY1341	*M. gryllidicola*
49	NHJ10822	*M. phasmatodea*
50	MY6900	*M. phasmatodea*
52	ARSEF2133, CBS700.74	*M. flavoviride*
53	ARSEF4124	*M. frigidum*
54	CBS130.71	*M. anisopliae sensu stricto*
55	ARSEF7412	*M. anisopliae sensu stricto*
61	ARSEF7488	*M. anisopliae sensu stricto*
56	ARSEF7486	*M. acridum*
57	ARSEF324	*M. acridum*
58	ARSEF8820	*M. robertsii*
59	ARSEF4739	*M. robertsii*
60	ARSEF727	*M. robertsii*
62	MY0008	*M. clavatum*
63	MY11677	*M. clavatum*
64	MY11637	*M. clavatum*
65	ARSEF1015, ARSEF1914	*M. majus*

Table S2. Statistical analyses (one-factor ANOVA) of morphological characters of *Metarhizium* spp. DF = degree of freedom (Statistical significance: * **=** p-value < 0.05, ** = p-value < 0.01, *** = p-value < 0.001)

Comparison	Traits	F statistic	DF	p-value
M. anisopliae sensu lato (*M. neoanisopliae* vs. *M. hybridum*)	Phialide Width	1.8712	1	0.230
	Phialide Length	0.5734	1	0.483
	Conidia Width	31.323	1	0.003**
	Conidia Length	0.0058	1	0.942
M. pingshaense sensu lato (*M. pingshaense* vs. *M. parapingshaense*)	Phialide Width	0.6899	1	0.438
	Phialide Length	6.4214	1	0.044*
	Conidia Width	0.0846	1	0.781
	Conidia Length	6.1598	1	0.095
	Conidia Width (specimen)	28.583	1	**8.676e-07*****
	Conidia Length (specimen)	96.174	1	**2.968e-15*****
M. anisopliae sensu stricto vs. *M. anisopliae sensu lato*	Phialide Width	0.0012	1	1.00
	Phialide Length	0.1393	1	1.00
	Conidia Width	0.4431	1	1.00
	Conidia Length	0.0192	1	0.980

Table S3. The least mean square contrasts between *Metarhizium* spp. within PARB group, estimated from the data of mortality with mycelia derived from the virulence assays against *Spodoptera exigua* (*Lepidoptera*). SE = Standard deviation. df = degree of freedom.

Contrast	estimate	SE	df	t.ratio	p.value
M. anisopliae – M. neoanisopliae	-0.45048	0.0413	251	- 10.903	**<.0001**
M. anisopliae – M. parapingshaense	-0.39048	0.0494	251	-7.907	**<.0001**
M. anisopliae – M. pingshaense	-0.39365	0.0451	251	-8.732	**<.0001**
M. anisopliae – M. robertsii	-0.3	0.0605	251	-4.96	**<.0001**
M. anisopliae – M. hybridum	-0.54286	0.0605	251	-8.975	**<.0001**
M. neoanisopliae – M. parapingshaense	0.06	0.0413	251	1.452	0.6949
M. neoanisopliae – M. pingshaense	0.05683	0.0361	251	1.576	0.6154
M. neoanisopliae – M. robertsii	0.15048	0.0541	251	2.782	0.0638
M. neoanisopliae – M. hybridum	-0.09238	0.0541	251	-1.708	0.528
M. parapingshaense – M. pingshaense	-0.00317	0.0451	251	-0.07	1
M. parapingshaense – M. robertsii	0.09048	0.0605	251	1.496	0.6672
M. parapingshaense – M. hybridum	-0.15238	0.0605	251	-2.519	0.1224
M. pingshaense – M. robertsii	0.09365	0.057	251	1.642	0.5713
M. pingshaense – M. hybridum	-0.14921	0.057	251	-2.617	0.097

M. robertsii – M. hybridum	-0.24286	0.0698	251	-3.477	0.0078

Table S4. The least mean square contrasts between *Metarhizium* spp. within PARB group, estimated from the data of unconditional mortality derived from the virulence assays against *Spodoptera exigua* (*Lepidoptera*). SE = standard deviation. df = degree of freedom.

**contrast**	**estimate**	**SE**	**df**	**t.ratio**	**p.value**
M. anisopliae – M. hybridum	-0.3929	0.065	272	-6.04	**<.0001**
M. anisopliae – M. neoanisopliae	-0.327	0.0434	272	-7.541	**<.0001**
M. anisopliae – M. parapingshaense	-0.219	0.0531	272	-4.125	**0.0007**
M. anisopliae – M. pingshaense	-0.296	0.0485	272	-6.107	**<.0001**
M. anisopliae – M. robertsii	-0.1786	0.065	272	-2.746	0.0698
M. hybridum – M. neoanisopliae	0.0659	0.0574	272	1.148	0.8605
M. hybridum – M. parapingshaense	0.1738	0.065	272	2.672	0.0842
M. hybridum – M. pingshaense	0.0968	0.0613	272	1.579	0.6131
M. hybridum – M. robertsii	0.2143	0.0751	272	2.853	0.0524
M. neoanisopliae – M. parapingshaense	0.1079	0.0434	272	2.489	0.1308
M. neoanisopliae – M. pingshaense	0.031	0.0375	272	0.824	0.9629
M. neoanisopliae – M. robertsii	0.1484	0.0574	272	2.588	0.1038
M. parapingshaense – M. pingshaense	-0.077	0.0485	272	-1.588	0.6071
M. parapingshaense – M. robertsii	0.0405	0.065	272	0.622	0.9893
M. pingshaense – M. robertsii	0.1175	0.0613	272	1.916	0.3948
