# Supplementary figures and images for: Integrative taxonomy of Metarhizium anisopliae species complex, based on phylogenomics combined with morphometrics, metabolomics, and virulence data
Source: IMA Fungus. 2024 Sep 11;15:30. doi: 10.1186/s43008-024-00154-9 (PMC11389511; doi:10.1186/s43008-024-00154-9)

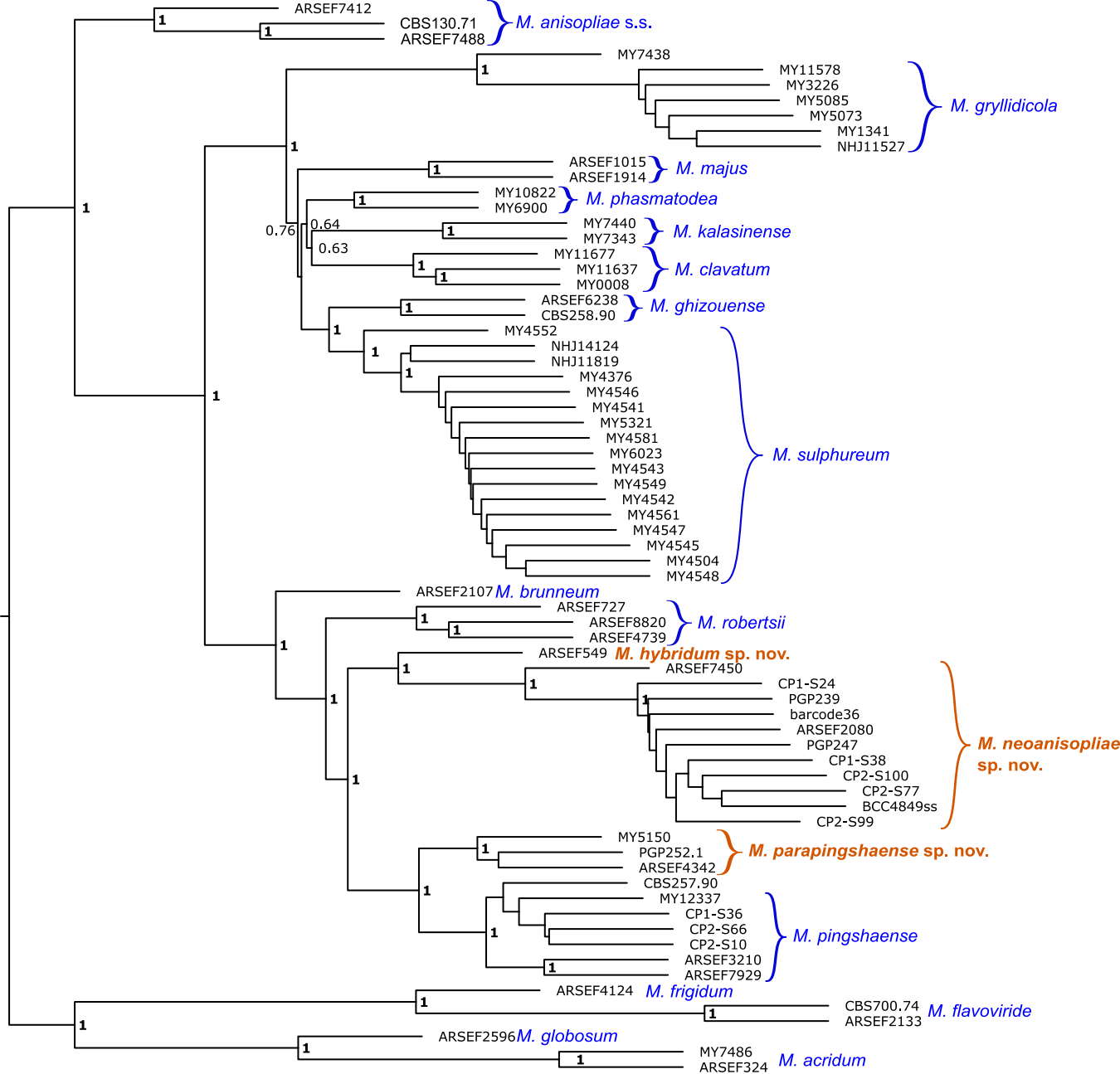

Supplement: Supplementary file 2 — Additional file 2: Figure S1. A coalescent-based species tree based on the reconciliation of 238 gene trees. The nodes are shown with values of posterior probability [file 43008_2024_154_MOESM2_ESM.pdf]

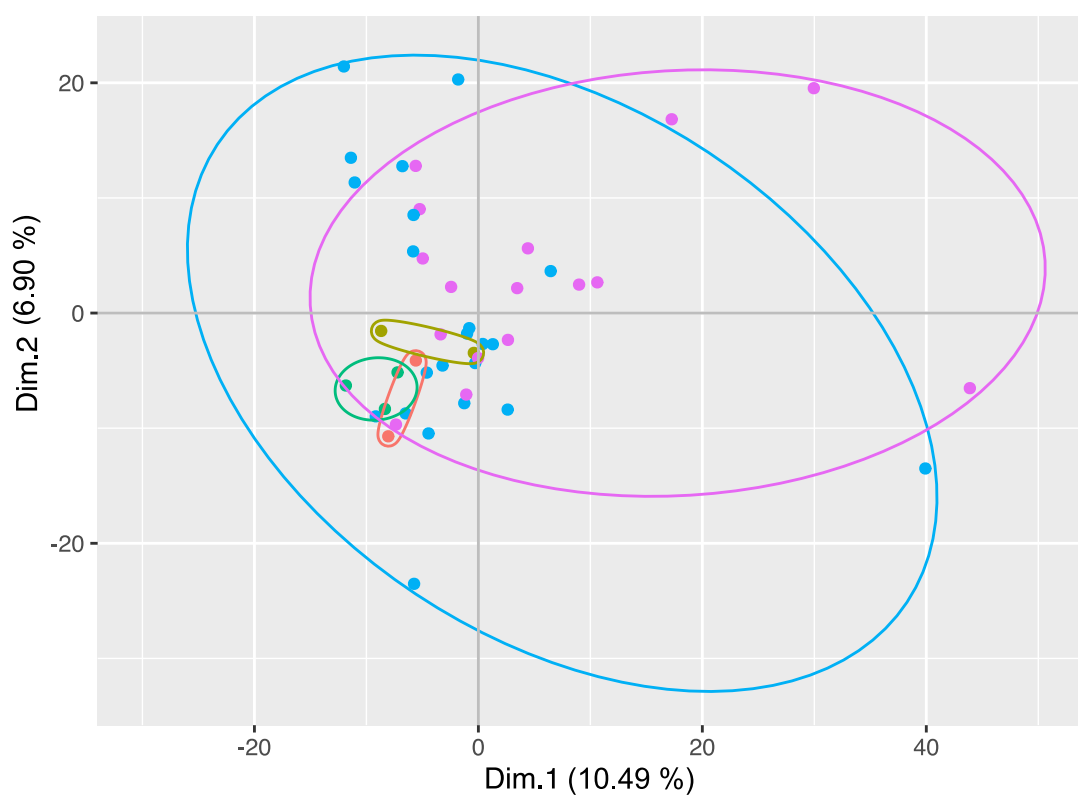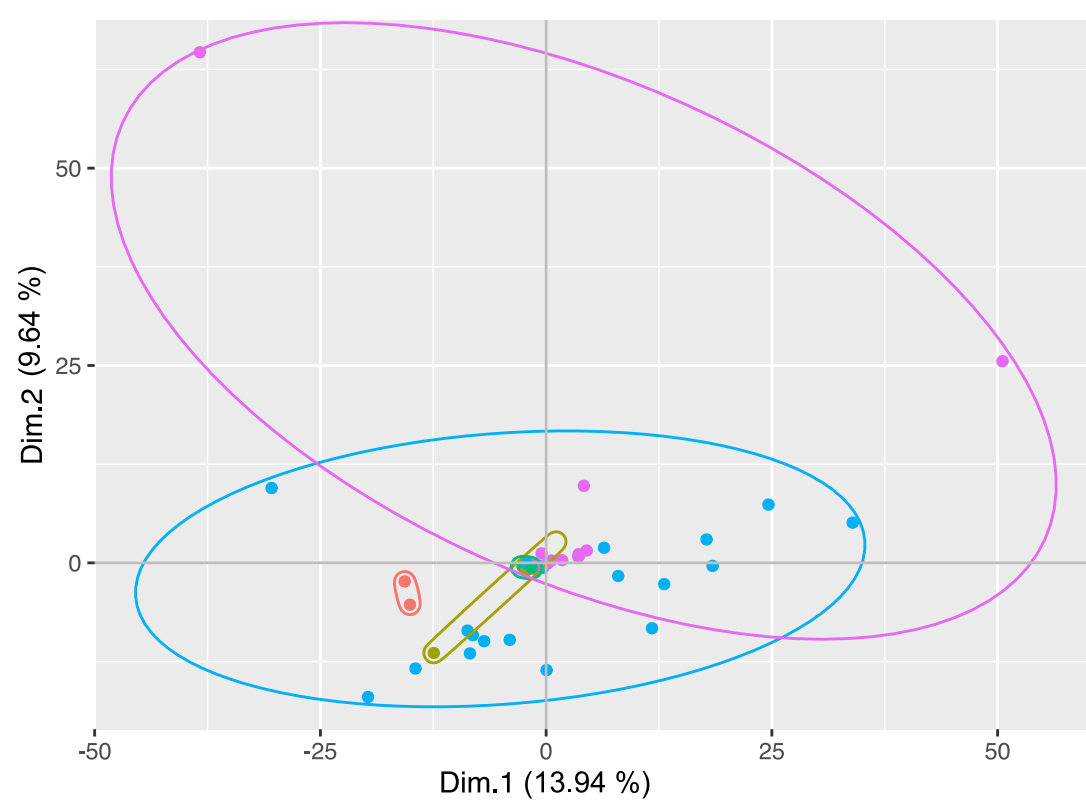

● Acridum ● Anisopliae strict ● Flavoviride ● MGT ● PARB

Supplement: Supplementary file 5 — Additional file 5: Figure S4. Metabolomic analyses based on peak area data obtained from liquid chromatography-mass spectrometry: comparison between different species complexes. Principal component analyses of data from cell extracts, and broth extracts. Acridum = M. acridum complex [M. acridum + M. globosum]; Anisopliae strict = M. anisopliae sensu stricto; Flavoviride = M. flavoviride complex [M. flavoviride + M. frigidum]; MGT = MGT group [M. majus, M. guizhouense, M. clavatum, M. gryllidicola, M. kalasinense, M. phasmatodea and M. sulphureum]; PARB = PARB group [M. brunneum, M. hybridum, M. neoanisopliae,M. parapingshaense, M. pingshaense]. [file 43008_2024_154_MOESM5_ESM.pdf]
