# Supplementary material for: Integrative taxonomy of Metarhizium anisopliae species complex, based on phylogenomics combined with morphometrics, metabolomics, and virulence data
Source: IMA Fungus. 2024 Sep 11;15:30. doi: 10.1186/s43008-024-00154-9 (PMC11389511; doi:10.1186/s43008-024-00154-9)
Supplement: Supplementary file 3 — Additional file 3: Figure S2. Intraspecific variation of virulence. Mortality with mycelia: only dead insects covered by fungal mycelia were counted.Unconditional mortality: all dead insects were counted. The error bars represent standard errors [file 43008_2024_154_MOESM3_ESM.pdf]

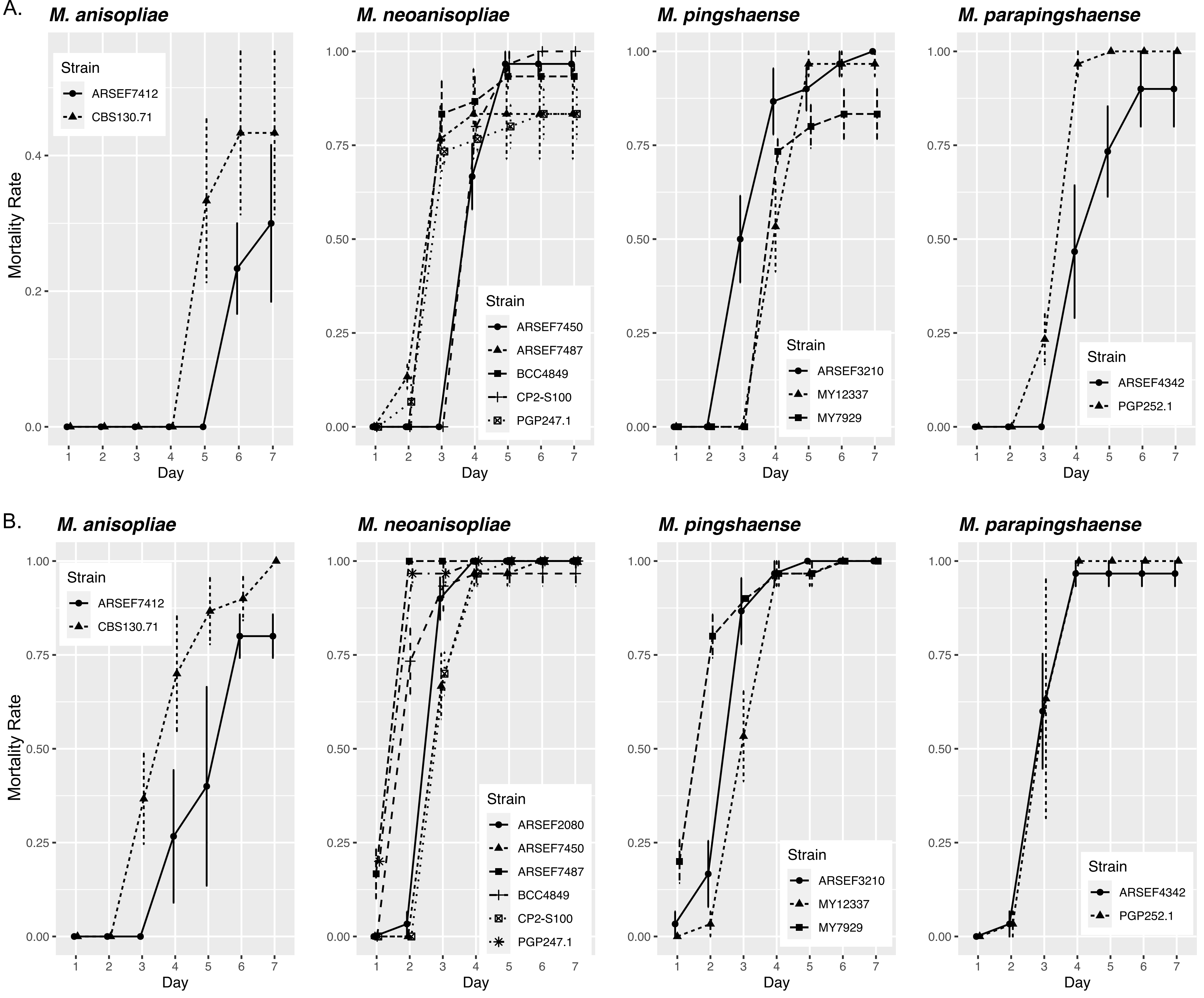
A.
M. anisopliae
M. neoanisopliae
M. pingshaense
M. parapingshaense
Strain
ARSEF7412
CBS130.71
ARSEF7450
ARSEF7487
BCC4849
CP2-S100
PGP247.1
ARSEF3210
MY12337
MY7929
ARSEF4342
PGP252.1
Mortality Rate
Day
B.
M. anisopliae
M. neoanisopliae
M. pingshaense
M. parapingshaense
Strain
ARSEF7412
CBS130.71
ARSEF2080
ARSEF7450
ARSEF7487
BCC4849
CP2-S100
PGP247.1
ARSEF3210
MY12337
MY7929
ARSEF4342
PGP252.1
Mortality Rate
Day
