# Supplementary material for: Integrative taxonomy of Metarhizium anisopliae species complex, based on phylogenomics combined with morphometrics, metabolomics, and virulence data
Source: IMA Fungus. 2024 Sep 11;15:30. doi: 10.1186/s43008-024-00154-9 (PMC11389511; doi:10.1186/s43008-024-00154-9)
Supplement: Supplementary file 4 — Additional file 4: Figure S3. Metabolomic analyses based on peak area data obtained from liquid chromatography-mass spectrometryfor all Metarhizium species included in this study. Principal component analyses of data from cell extracts, and broth extracts. [file 43008_2024_154_MOESM4_ESM.pdf]

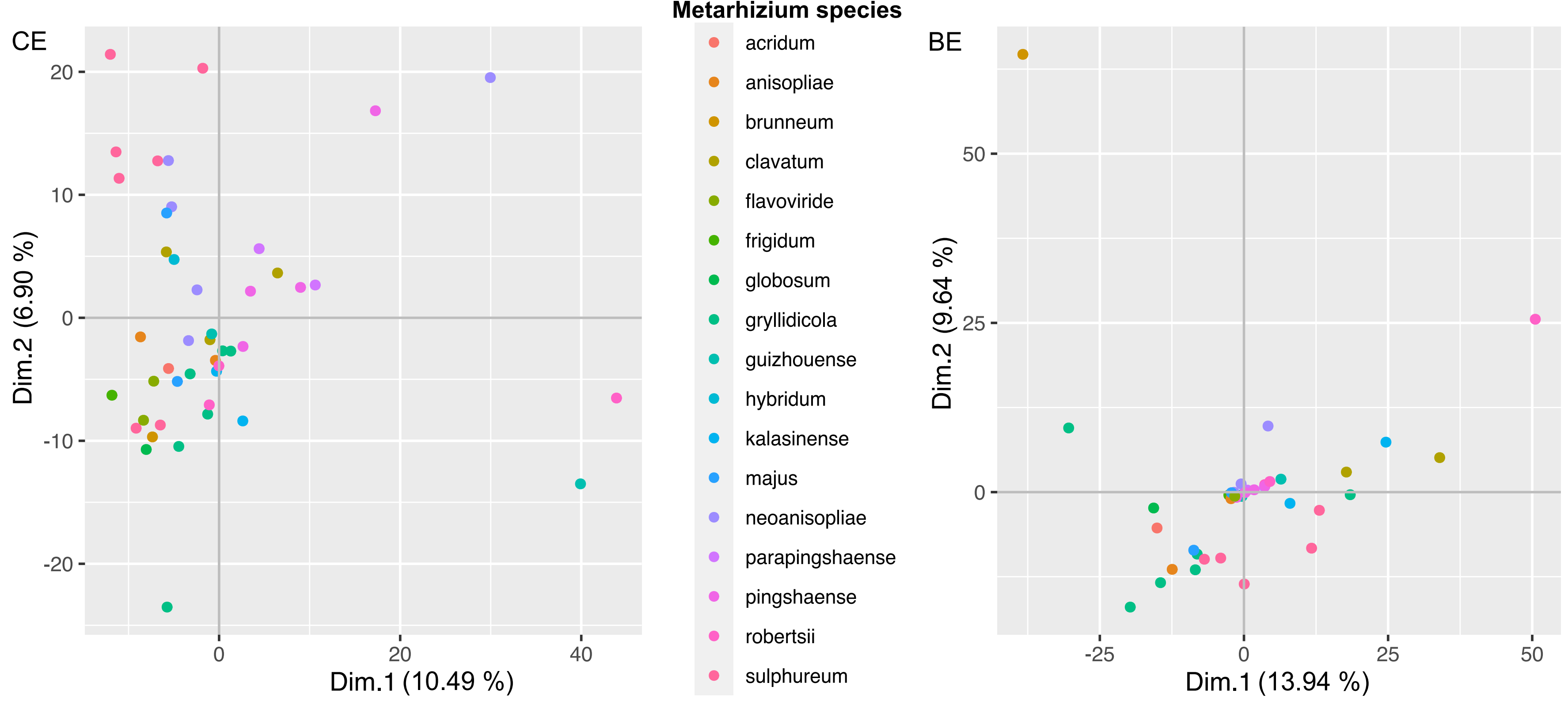

CE
Dim.2 (6.90 %)
Dim.1 (10.49 %)
20
10
0
-10
-20
0
20
40
Metarhizium species
acridum
anisopliae
brunneum
clavatum
flavoviride
frigidum
globosum
gryllidicola
guizhouense
hybridum
kalasinense
majus
neoanisopliae
parapingshaense
pingshaense
robertsii
sulphureum
BE
Dim.2 (9.64 %)
Dim.1 (13.94 %)
50
25
0
-25
0
25
50
